# Melatonin seed priming reconfigures the metabolomic profile and functional attributes of brown mustard (*Brassica juncea L.)* sprouts

**DOI:** 10.1038/s41598-026-68267-2

**Published:** 2026-09-03

**Authors:** Dina S. Ghallab, Tarek M. Okda

**Affiliations:** 1 Department of Pharmacognosy, Faculty of Pharmacy, Alexandria University, Alkhartoom Square, Alexandria, 21521 Egypt; 2 Department of Biochemistry, Faculty of Pharmacy, Damanhour University, Damanhour, Egypt

**Keywords:** Melatonin priming, Germination, Mustard seeds, KEGG pathway analysis, Alzheimer’s disease, Biochemistry, Biotechnology, Chemical biology, Plant sciences

## Abstract

While mustard seeds offer a prime store of biologically active compounds, there is insufficient data regarding the impact of melatonin (MT) priming on remodelling the chemical patterns, metabolic pathways, and biological attributes of mustard sprouts. Accordingly, the current work is directed to investigate the metabolic shifts in mustard sprouts post MT-priming treatments using UPLC–MS/MS and chemometric analyses. Given this, MT-primed sprouted samples witnessed a significant elevation of phenolic acids, specifically hydroxybenzoic, ferulic, syringic, and chlorogenic acids, with approximately 2.5- to 6-fold increments compared to unprimed controls. In MT-primed mustard sprouts, some aliphatic glucosinolates, such as gluconapin, glucotropaeolin, and sinigrin, revealed a notable elevation with fold increments of 3.5, 2.9, and 2.6, respectively, compared to unprimed counterparts. Further, some flavonoids and stilbenes, primarily isorhamnetin, syringetin-O-glucoside, quercetin, and isorhapontigenin, exhibited a notable enrichment pattern in primed mustard sprouts relative to the others. KEGG pathway analysis revealed phenylpropanoid biosynthesis, flavonoid biosynthesis, glucosinolate biosynthesis, and stilbenoid, diarylheptanoid and gingerol biosynthesis pathways as the key metabolic pathways primarily govern these metabolic fluctuations. Regarding AChE and MAO-B inhibitory screening, MT-primed sprouted samples recorded the most leading observations with IC_50_ = 7.667 ± 0.306 mg/dl and 45.389 ± 0.953 mg/dl, respectively. OPLS-driven correlation analysis pinpointed syringic, ellagic, and chlorogenic acid, along with quercetin, pterostilbene, sinigrin, gluconapin, and eicosapentaenoic acid, majorly detected in MT-primed samples, as biologically active compounds beyond their AChE and MAO-B inhibitory activities. These findings support melatonin priming as a sustainable, effective technique for creating high-quality, nutritious, and health-promoting agricultural products to rectify chronic diseases.

**Supplementary Information:**

The online version contains supplementary material available at https://doi.org/10.1038/s41598-026-68267-2.

## Introduction

Alzheimer’s disease (AD) is a disease of a multifaceted nature and insidious onset, typically manifested by behavioral abnormalities, cognitive decline, and neuropsychiatric conditions that adversely affect one’s regular multitasking abilities^1,2^. Approximately 50 million individuals worldwide suffer from AD, and by 2050, this number is predicted to triple^3^. Currently, there is no specific therapy available, and medical management is primarily supportive. Acetylcholinesterase (AChE) inhibitors, such as donepezil and physostigmine are among the main drugs used for AD; however, none of these remedies have been shown to be a reliable treatment yet^4^. Additionally, one of the critical issues with the treatment in the clinic setting is the adverse medications’ side effects^5^. Given this situation, research on safer and more effective alternative treatments to reverse disease flare-ups is highly recommended. Being a traditional Chinese medicine with promising biological features, mustard seeds may be a decent nutraceutical option to combat such neurological issues quickly spread around the globe^6^.

Brown mustard seeds (*Brassica juncea* L.), part of the *Brassicaceae* family, are extensively grown and have a long history of use^7^. They are traditionally consumed for producing edible oils and condiments and are also key ingredients in sauces and fermented vegetable products^7,8^. In various Asian cultures, black, brown (oriental), and yellow (white) mustard seeds are established staples in both traditional diets and holistic medicine^8^. These seeds are frequently utilized as natural remedies to alleviate respiratory issues like colds and coughs, as well as to manage chronic conditions such as arthritis, muscle pain, and diabetes^9,10^. Chemically, mustard seeds are packed with bioactive constituents including glucosinolates, flavonoids, anthocyanins, phenolic acids, omega-3 fatty acids, and various sulphur-based compounds^7^. These phytochemicals are strongly linked to diverse therapeutic effects, including anticancer, antimicrobial, anti-inflammatory, and antiviral properties, as well as benefits for managing diabetes, neurological health, and blood clotting^8,11^. While multiple studies show mustard seeds are packed with bioactive compounds with significant therapeutic potential, they remain overlooked for nutraceutical applications especially in cognitive disorders, a potential area ripe for further exploration.

As urbanization and globalization accelerate, new techniques for processing edible seeds have emerged to enhance their nutritional and functional profiles^12,13^. These treatments aim to improve the digestibility of essential macro- and micro-nutrients while simultaneously reducing anti-nutritional factors that hinder absorption^13^. Aligning with the principles of nutritional therapy, germination is recognized as an auspicious green modification approach for enhancing valuable health-benefiting compounds in seeds, increasing the bioavailability and digestibility of key nutrients, and converting dormant seeds into functional foods^14^. In the same context, seed priming is a sustainable eco-friendly technique where seeds are immersed in a liquid containing beneficial biological agents like bacteria or fungi and/or natural substances for controlled hydration and initiation of pre-germination metabolic activities^15–17^. This preliminary step allows for faster germination, nutrient mobilization, bioactives accumulation, and better stress resilience^18^. Seed priming with melatonin (MT) serves as a straightforward and effective agricultural technique for boosting germination and seedling vigor, enhancing stress tolerance, and activating antioxidant defence enzymes, promoting crop yield and quality even in stressful conditions^19^. Melatonin primes seeds by activating enzymatic machinery and initiating various metabolic activities before sprouting, which in turn modulates the production of protective bioactive compounds^20^. In parallel with the literature, a recent seminal study has proved that melatonin-seed priming effectively alleviates environmental stress conditions through activating antioxidant defense enzymes, mainly superoxide dismutase (SOD), catalase (CAT), glutathione peroxidase (GPX), and ascorbate peroxidase (APX), in maize seeds^21^. This treatment also improved soluble sugar, proline, and chlorophyll content in the seeds^21^.

Relatedly, it has been reported that exogenous application of melatonin enhances bean germination qualities and elevating pigments, phenolic content, sugars, indole acetic acid, calcium, zinc, potassium, vitamin E, and C^22^. Another study demonstrated that that seed priming with melatonin improves germination and seedling resilience under drought and water-deficit conditions by boosting antioxidant enzyme activities, maintaining cellular water status, and regulating stress-response hormones^23^. It also regulated the release of key plant hormones, augmenting gibberellic acid (GA₃) content and decreasing abscisic acid (ABA) content^24^. A recent interesting report showed that priming with 50 µM melatonin improved early growth, shoot length, and dry weight in tomato seedlings under severe chilling conditions (12 °C)^20^. This treatment significantly reduced oxidative stress markers like malondialdehyde (MDA) and hydrogen peroxide while increasing total polyphenols and flavonoids^20^. These studies have primarily proved that melatonin serves as a promising antioxidant and a master regulator of plant physiological and metabolic processes.

while previous studies have emphasized on the positive impact of melatonin in enhancing seed germination and seedling growth, as well as improving tolerance to various abiotic stresses (drought and chilling), some research gaps remain. Specifically, there is a need to investigate the metabolic dynamics and spatial distribution of bioactive compounds following seed biopriming with melatonin as a physiological bio-stimulant to optimize the yield of health-promoting bioactive compounds through resource-efficient production methods. Also, pinpointing key molecular signalling pathways associated with melatonin seed priming impact has been underexplored till now.

To fill these gaps, the study employed an integrated approach, utilizing Ultra-high Performance Liquid Chromatography coupled with mass spectrometry (UPLC–MS) metabolomics and subsequent multivariate data analysis to offer a detailed qualitative and quantitative description of the unique chemical profiles (metabolomes) of mustard seeds preliminarily primed with melatonin during early seed germination phase. Concurrently, demonstrating the regulatory metabolic pathways that govern the observing chemical shifts in analysed melatonin-primed seeds was investigated and traced. To experimentally validate the efficacy of melatonin priming in enhancing bioactive compound in mustard seeds for AD control, the MT-primed samples were directly evaluated for their inhibitory potential against acetylcholinesterase (AChE) and monoamine oxidase (MAO-B) enzymes. Complementarily, bioinformatics tools were utilized to assign the efficacy chemical signatures that work in synergy to target and mange AD. Notably, no prior study has explored how melatonin treatment as a seed bio-priming agent influences the chemical makeup and function of mustard seeds throughout the seedling growth phase. Such integrative research bridges knowledge gaps regarding how melatonin influences mustard seed physiology, chemistry, metabolic pathways, and biological traits during germination. These findings support using melatonin priming as a sustainable effective strategy to enhance global crop yields and develop high-value nutraceutical products.

## Materials and methods

### Chemicals and reagents

Melatonin (MT; $\:\ge\:$98% purity), authentic commercial standards (sinigrin, quercetin, ferulic acid, stigmasterol, and leucine), assay kits for determining AChE and MAO-B content as well as physostigmine and pargyline (positive control) were supplied by Sigma-Aldrich (St Louis, MO, USA). Methanol, acetonitrile and formic acid utilized for LC–MS analysis were of chromatographic grade and obtained from Merck, Darmstadt, Germany.

### Seeds preparation and study design

As a continuation to our recently published report^25^, the uniform brown mustard seeds (50 g) obtained from the Agricultural Research Centre at Cairo University in Giza, Egypt and subsequently surface-disinfected using a 0.5% sodium hypochlorite solution were subjected to melatonin priming to investigate the superior effect of melatonin as a biostimulant on the chemical profile and biological properties. Of note, to provide a baseline for comparison and assure the experiment’s reliability, 50 g of mustard seeds was subjected to germination without pre-sowing treatment with melatonin, acting as a negative control or comparison set and labelled with MS (G) (germinated mustard seeds, unprimed control sample).

Given this, the primed germinated portion of mustard seeds was compared with germinated unprimed one. The detailed description of melatonin priming process was delineated in the following section.

### Melatonin preparation and seed priming procedure prior germination process

Melatonin stock solutions (10 mM) were prepared by dissolving pure melatonin powder in 1 mL of dimethyl sulfoxide (DMSO) which is then gently brought to a final volume of 10 mL using sterile distilled water. After that, a standard 100 µM working solution is prepared by mixing 1.0 mL of the 10 mM stock into 99.0 mL of sterile distilled water. To eliminate solvent bias, the hydropriming control solution contained an equivalent concentration of DMSO ($\:0.1\%$ v/v in $\:{\mathrm{ddH}}_{2}\mathrm{O}$) was prepared where unprimed seeds served as a control.

Based on previous studies^26,27^, 100 µM was selected as the optimal melatonin (MT) priming concentration for enhancing the germination rate.

Practically speaking, 50 g of healthy, uniform seeds of brown mustard (10 g per replicate) were surface-sterilized in 1% (v/v) sodium hypochlorite for 3 min and thoroughly rinsed three times with sterile $\:{\mathrm{ddH}}_{2}\mathrm{O}$. Seeds were then fully immersed in their respective priming solutions at a seed-to-volume ratio of 1:5 (w/v; 10 g seeds per 50 mL solution) in amber glass vessels to prevent melatonin photodegradation. Priming was carried out at $\:25\pm\:1$ °C under continuous dark conditions for 12 h. Following priming, seeds were gently rinsed with distilled water to remove surface-bound residues, surface-blotted with filter paper, and re-dried in a climate chamber (* 25* °C, $\:45\%$ relative humidity). The re-dried seeds were then subjected to ten-day germination process following our previous protocols with minor adjustments^28^. After that, the primed germinated seeds were lyophilized, ground, labelled with MS (MT) (MT-primed germinated mustard samples), and kept at 4 °C until further analysis.

Next, the gathered portions of mustard seeds (MT-primed and unprimed germinated) were individually submitted to the extraction process aligning with our previously optimized method^29^. Briefly, the resulting fractions were extracted in duplicate using a 3 L Alpha Plus ultrasonic bath (Japan) with 200 mL of 70% ethanol at 40 °C for 60 min. The yielded extract was subsequently concentrated to dryness via vacuum evaporation utilizing a Buchi Rotavapor (Switzerland). Five independent replicates for the primed fraction were prepared for phytochemical investigation.

### Phytochemical investigation of sprouted mustard extracts using UPLC-QqQ-MS/MS

Chemical profiling of sprouted mustard extracts was carried out using a Shimadzu 8045 UPLC triple quadrupole (QqQ) apparatus (Shimadzu Corporation, Kyoto, Japan) integrated with an electrospray ionization (ESI) source.

The Supplementary Material provides a detailed breakdown of the chromatographic conditions, ESI interface settings, and mass data processing parameters, all conducted according to^30,31^ protocols.

### Multivariate statistical analysis and metabolic pathway analysis

To perform chemometric analysis, the raw UPLC-MS data were processed using SIMCA-P software (version 14.1). Principal Component Analysis (PCA) was initially employed to visualize the dataset, identifying general trends, clusters, and outliers. Subsequently, with a scope to catch differential metabolites associated with melatonin priming during mustard seed germination, OPLS-DA model was implemented with a pairwise comparison approach between germinated unprimed samples and MT-primed germinated ones (MS (G) & MS (MT)). In OPLS-DA, metabolites with VIP (Variable Importance in Projection) values exceeding 1 were considered to have notably significant contributions to the observed group discrimination. A one-way analysis of variance (ANOVA) and subsequent Tukey’s multiple range test were employed to assess the statistical differences in concentration means among various mustard samples. Aligning with previous investigations^32,33^, significance threshold of *p* < 0.05 and fold-change (FC) > 2.0 (or < 0.5) were used where metabolites were deemed “differential metabolites” if they satisfied the criteria of a VIP score greater than 1, an FC greater than 2.0 (or less than 0.5), and *p* < 0.05. further, the predictive reliability of the established models was evaluated using the corresponding R^2^ and Q^2^ values.

MetaboAnalyst 6.0 (https://www.metaboanalyst.ca/) was utilized for metabolic pathway enrichment analysis using the Kyoto Encyclopedia of Genes and Genomes (KEGG) database^34,35^, assigning the key metabolic pathways linked with differential metabolites between the germinated (unprimed) and MT-primed germinated samples. Of note, significant metabolic pathways were defined as those with a *P* value below 0.05 and an impact score exceeding 0.1^36,37^.

### Screening of acetylcholine esterase and monoamine oxidase inhibitory activities of the different mustard extracts

In an effort to experimentally validate the positive effect of melatonin priming on bioactive compounds accumulation and biological properties enhancement, the raw, germinated (unprimed), and MT-primed germinated mustard samples were subjected to in vitro screening for acetylcholine esterase (AChE) and monoamine oxidase (MAO-B) inhibitory activities following^38,39^ methods with minor adjustments.

#### Acetylcholine esterase (AChE) inhibitory activity

The inhibitory activity against acetylcholinesterase (AChE) was assessed using a colorimetric method adapted from the classical Ellman assay^38^ performed with a commercially available Acetylcholinesterase Inhibitor Screening Kit (MAK324, Sigma-Aldrich, USA). This assay is based on the enzymatic hydrolysis of acetylthiocholine by AChE, producing thiocholine, which subsequently reacts with 5,5′-dithiobis (2-nitrobenzoic acid) (DTNB) to generate a yellow-colored product measurable at 412 nm. The assay was conducted in clear, flat-bottom 96-well microplates. Purified acetylcholinesterase was prepared in assay buffer (pH 7.5) at a final concentration of 400 U/L, as recommended for reliable enzyme kinetics^40^. Test extracts at various concentrations (10, 25, 50, 100, 150, and 200 mg/dL) were dissolved in an appropriate solvent, ensuring that the final solvent concentration remained within the tolerated range for enzyme activity. The enzyme was pre-incubated with the test extracts for 15 min at room temperature to allow sufficient enzyme–inhibitor interaction. The reaction was initiated by adding a freshly prepared reaction mixture containing assay buffer, acetylthiocholine substrate, and DTNB. Absorbance at 412 nm was recorded immediately after reaction initiation and after 10 min using a microplate reader. Wells containing enzyme without inhibitors served as negative controls, while enzyme-free wells were used for background correction. AChE inhibition was calculated as the percentage decrease in enzyme activity relative to the untreated control, based on the change in absorbance over time. All experiments were performed in triplicate, and results were expressed as mean ± standard deviation.

#### Monoamine oxidase inhibitory activity

Monoamine oxidase (MAO) inhibitory activity was determined using a sensitive fluorometric assay based on hydrogen peroxide generation during monoamine oxidation, employing a commercial Monoamine Oxidase Inhibitor Screening Kit (Catalogue No. BA0188, AssayGenie, Ireland). This method relies on the enzymatic oxidative deamination of p-tyramine, a non-selective substrate for both MAO-A and MAO-B isoforms, leading to the formation of hydrogen peroxide, which is subsequently detected through a horseradish peroxidase–coupled fluorescent reaction^39^. All experiments were carried out in black, flat-bottom 96-well microplates to minimize background fluorescence. Purified human MAO-A and MAO-B enzymes were prepared at final activities of 3 U/mL and 6 U/mL, respectively, in assay buffer (pH 7.4), as previously described for optimal kinetic performance^41^. Test extracts were dissolved in an appropriate solvent, and the final solvent concentration was maintained within the enzyme tolerance range. Enzymes were pre-incubated with test compounds for 15 min at 25 °C to allow sufficient inhibitor–enzyme interaction.

The enzymatic reaction was initiated by the addition of freshly prepared working reagent containing assay buffer, diluted p-tyramine substrate, dye reagent, and horseradish peroxidase. Following incubation for 20 min in the dark, fluorescence intensity was measured at an excitation wavelength of 530 nm and an emission wavelength of 585 nm using a microplate fluorescence reader. Wells lacking substrate were used as blanks, while enzyme-containing wells without inhibitors served as negative controls. Pargyline was set as a positive control to validate assay performance. MAO activity was calculated after background subtraction and expressed as a percentage relative to untreated controls. All measurements were performed in triplicate, and data were reported as mean ± standard deviation.

Orthogonal projection to latent structures (OPLS) was subsequently utilized as a biologically focused classification tool to identify the primary active constituents responsible for the AChE and MOA inhibition observed in the mustard extracts. The reliability of these models was assessed using cumulative R^2^ and Q^2^ metrics, with values approaching 1 signifying superior predictive performance and accuracy.

### Statistical analysis

The data is presented as means ± standard deviations (SD). To compare the groups, a one-way analysis of variance (ANOVA) followed by the Tukey post hoc test were performed using Graph Pad InStat Software Inc., program, version 8.1.0 Philadelphia, San Diego CA, USA. A *p*-value of less than 0.05 was used as the threshold for determining statistical significance.

## Results and discussion

### Phytochemical investigation of mustard sprouts preliminarily primed with melatonin

As a continuation to our recently published report^25^ and with a scope to enhance bioactive compounds in the mustard samples, melatonin as a biostimulant was preliminarily applied on the mustard seeds prior germination process. Given this, UPLC-MS/MS metabolomics study was implemented to qualitatively and quantitatively monitor the metabolic signatures associated with MT-primed mustard sprouts compared to unprimed matches. Table S1 outlines the chemical compounds found in the raw, germinated (unprimed), and MT-primed germinated mustard samples, detailing their structural characteristics including retention time, parent and fragment ions, chemical classes, and Human Metabolome Database (HMDB) ID. Also, the base peak chromatograms (BPCs) for unprimed germinated (control) and MT-primed mustard samples, captured in both positive-ion and negative-ion modes were depicted in Fig S1.

Regarding metabolite annotation and aligning with Metabolomics Standards Initiative (MSI) guidelines, the 71 chromatographic signals embracing various chemical classes primarily glucosinolates, flavonoids, terpenoids, phenolic and fatty acids were chemically annotated by comparing the collected mass data against external standards, self-created database, publicly available spectral databases such as METLIN, mzCloud, and relevant scientific literature.

**Fig. 1:**
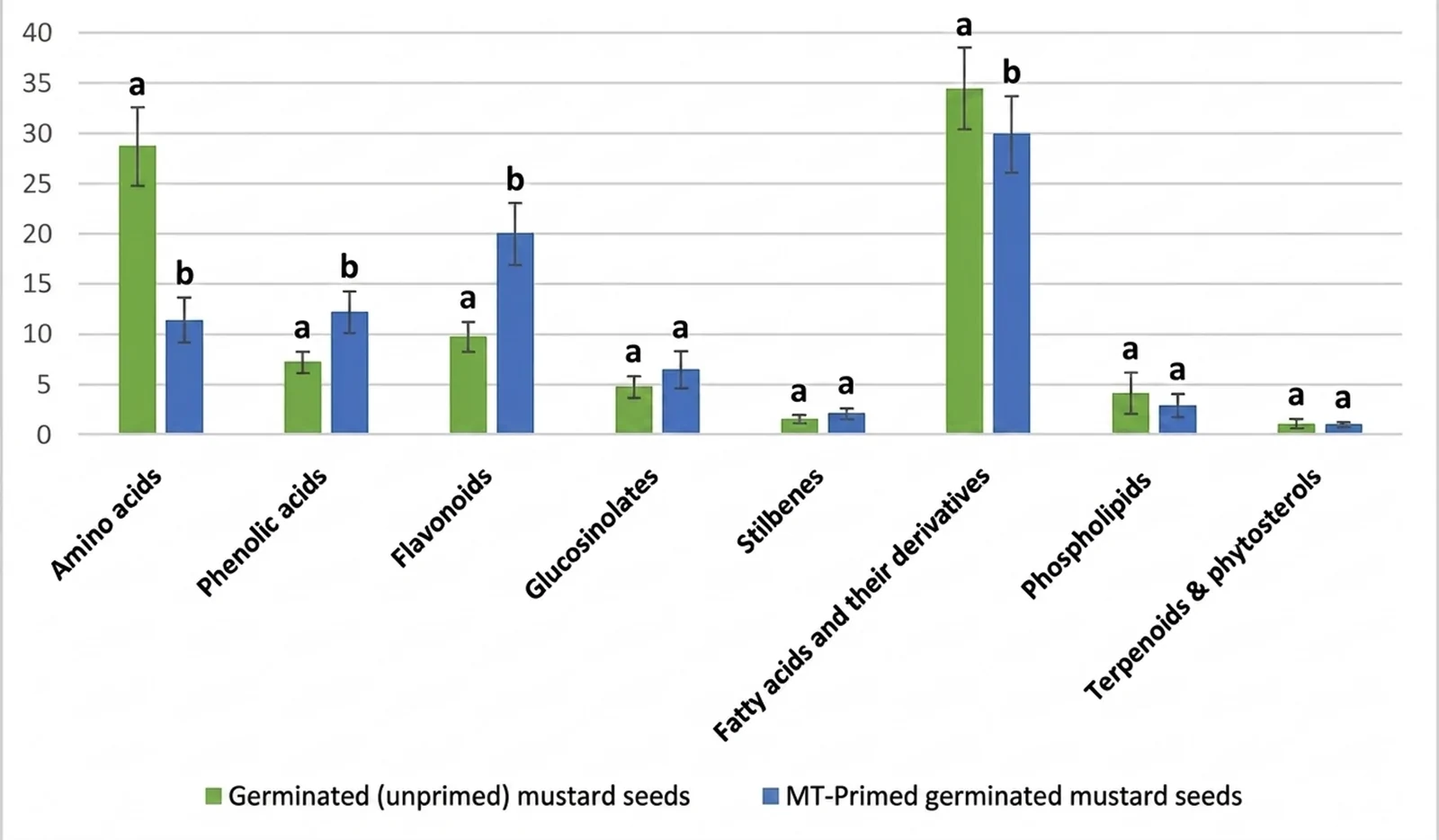
Bar charts visually illustrating the dynamic changes in the distribution of major chemical classes among unprimed and MT-primed mustard sprout samples. Note: Letters above bars denote statistical groups. Within each category, bars with different letters differ significantly (*p* < 0.05); bars with the same letter do not.

For clarity, the bar charts in Fig. 1 visualize the chemical makeup and distribution of the main chemical classes across the germinated (unprimed) and MT-primed mustard samples.

### Chemical discrimination of mustard sprouts following melatonin priming using multivariate statistical analysis

**Fig. 2:**
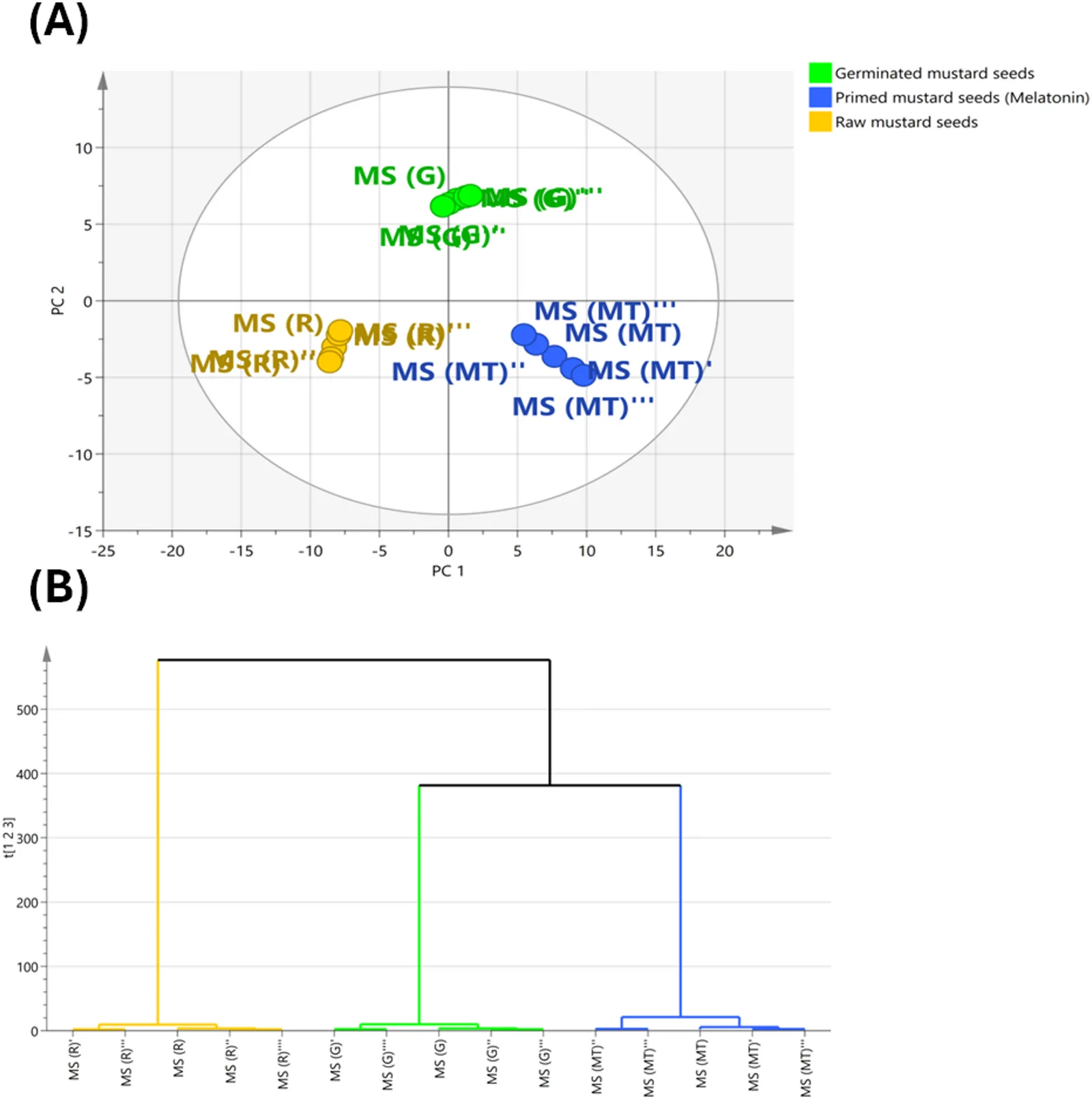
**(A)** PCA score scatter plot of different mustard samples (raw seeds, unprimed germinated and MT-primed germinated samples), (**B**) The corresponding dendrogram of hierarchical cluster analysis (HCA) by Ward algorithm.

To manage the multidimensional data generated by UPLC-MS, Principal Component Analysis (PCA) was utilized as an initial unsupervised exploratory tool, offering fundamental insights into sample distribution, variance, and clustering patterns across the PCA score plot. The three-component PCA model successfully divided the mustard samples into two primary clusters through the first two orthogonal PCs, which accounted for 77.3% of the total variance, demonstrating notable variation in their chemical composition (Fig. 2A). Specifically, germinated mustard samples (unprimed and MT-primed) showed clear chemical distinctions, evidenced by their unambiguous grouping along the positive direction of the PC1 axis, separate from the raw seed samples. PC2 distinguishes between unprimed and MT-primed mustard sprouts based on their inherent chemical differences, which are highlighted by their opposite positions along the PC2 axis (Fig. 2A). In other words, MT-priming caused a significant shifting in chemical composition compared to unprimed samples as primarily captured by PC2. Relatedly, Hierarchical Cluster Analysis (HCA), an unsupervised divisive tool, was conducted to provide a more intuitive overview of mustard samples clustering. Aligned with PCA results and as observed in the resulting dendrogram (Fig. 2B), the mustard samples were categorized into two primary clusters: one for raw seed samples, marking them as chemically different from the remaining germinated ones (unprimed and MT-primed) which are grouped in the second cluster. Within the second cluster, MT-primed germinated mustard samples formed a separate sub-cluster, showing measurable chemical differences compared to unprimed sprouted ones.

**Fig. 3:**
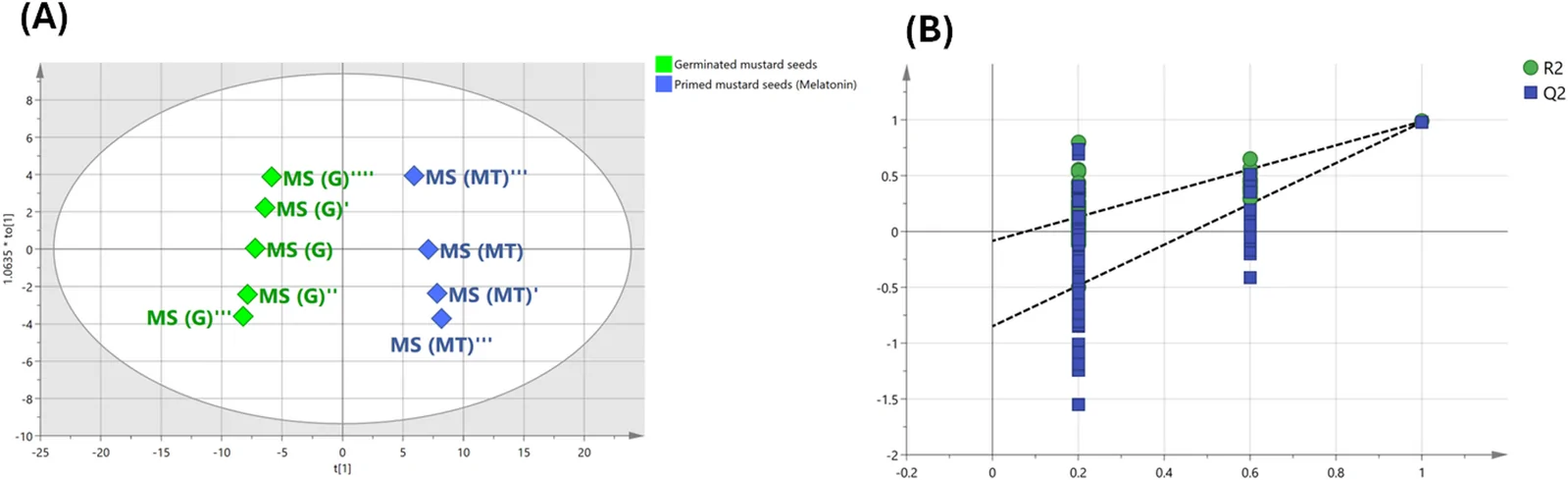
**(A)** A supervised OPLS-DA model in a pairwise comparison between the unprimed and MT-primed mustard sprout samples (MS (G) & MS (MT)), **(B)** permutation test (*n* = 200) for the established OPLS-DA.

To better understand the distinct metabolic shifts observed in mustard sprouts after MT-priming, a supervised OPLS-DA model (Fig. 3A) in a pairwise comparison between the unprimed and MT-primed mustard sprout samples (MS (G) & MS (MT)) was established, demonstrating high reliability and precision with performance parameters of R^2^X = 0.987, R^2^Y = 0.974, and Q^2^ = 0.96 scores. Additionally, permutation test (*n* = 200) established that the OPLS-DA model was not overfitted (Fig. 3B). Equally important, cross-validation (CV-ANOVA) further confirmed the overall statistical significance of the model (* F=55.55*, * p=0.00024*), validating that the metabolomic separation between the study groups is robust and highly significant.

### Metabolic trends and pathway analysis associated with melatonin priming and germination of mustard seeds

As stated above, melatonin acts as an effective biostimulant and edible seed priming with it in optimal concentrations significantly improves germination, growth, and stress tolerance^20^. However, no obtainable data focused on the dual impact of MT-priming and sprouting in tuning the chemical patterns and biological features of edible seeds, transforming dormant edible seeds into functional foods. Also, understanding the regulatory pathways which trigger complex biochemical changes in dormant seeds, fortifying their nutritional profile and bioavailability of various health-promoting bioactive compounds remains underexplored.

**Fig. 4:**
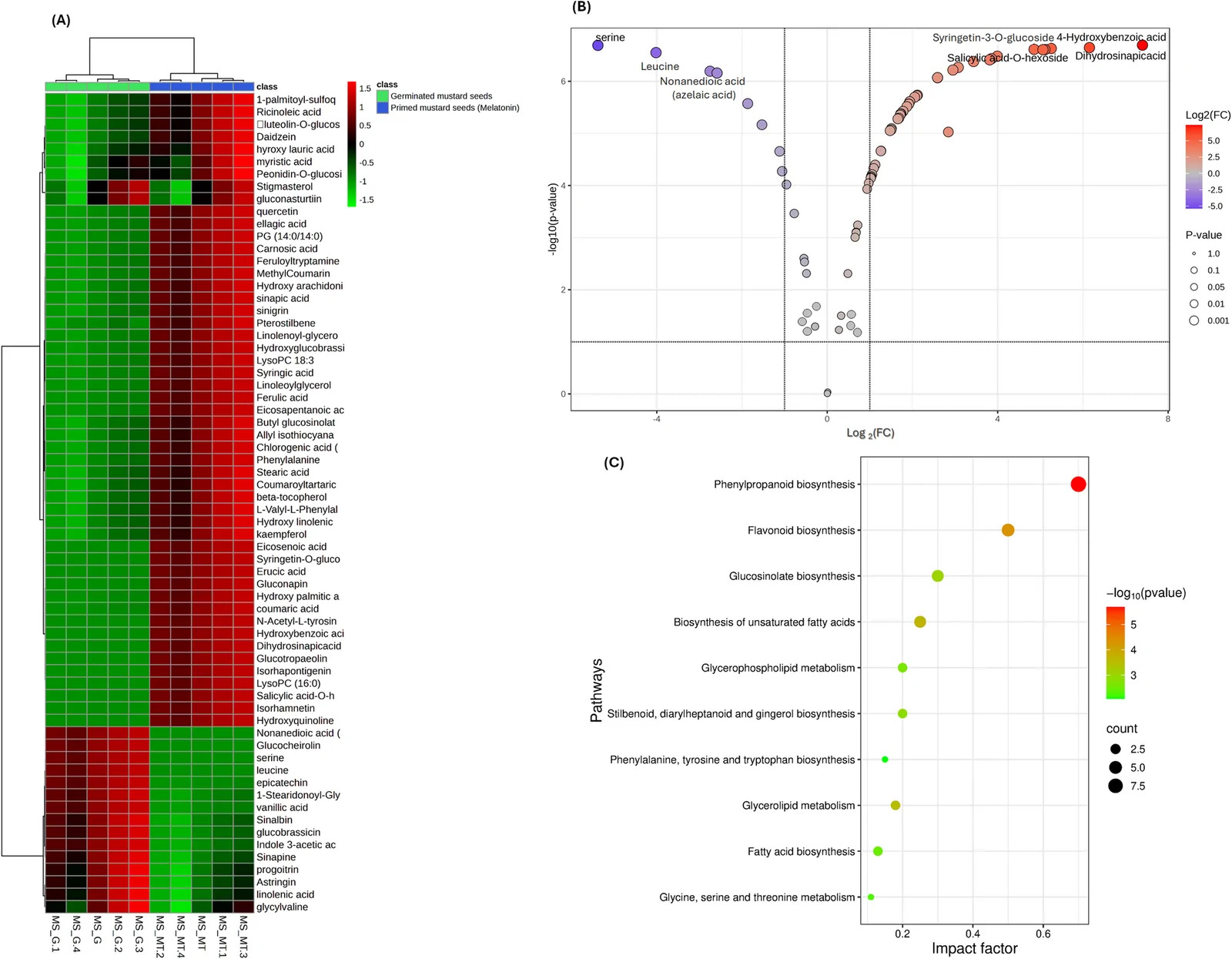
**(A)** A pairwise heatmap with a red-to-green colour gradient indicating high-to-low compound concentrations in unprimed and MT-primed mustard sprout samples, **(B)** The respective volcano plot, pinpointing the significantly altered metabolites based on a *p-*value < 0.05, a Variable Importance in Projection (VIP) score > 1, and a fold change (FC) exceeding 2 or falling below 0.5, where red dots represent significant up-regulation while purple ones indicate significant down-regulation of MS (MT)/MS (G), **(C)** KEGG enrichment analysis for the differential metabolites of the pairwise comparison; MS (MT)/MS (G).

To steadily visualize the fluctuations in metabolite levels between the unprimed and MT-primed mustard sprouts, a pairwise heatmap (Fig. 4A) with a red-to-green colour gradient indicating high-to-low concentrations was created. Further, a respective volcano plot (Fig. 4B) was inserted, pinpointing the significantly altered metabolites based on a *p-*value < 0.05, a Variable Importance in Projection (VIP) score > 1, and a fold change (FC) exceeding 2 or falling below 0.5. of note, the relative abundances across the treatment groups calculated from the raw replicate data (* n=5* for both groups), is depicted in Table S2.

To conduct metabolic pathway enrichment analysis, the differential metabolites were uploaded to MetaboAnalyst 6.0 and cross-referenced with KEGG database. Table 1 collectively outline the main metabolic differences between comparison samples (unprimed and MT-primed mustard sprouted samples), addressing VIP, FC, and *P* values, along with metabolic trends and respective regulatory pathways. The following subsections will detail the changes in primary metabolites and the related metabolic pathways observed in MT-primed mustard sprouts compared to unprimed sprouted ones.

**Table 1:** List of differential metabolites between unprimed and MT-primed mustard sprouts.

Compound name	VIP	$\:{\mathbf{log}}_{2}\left(\mathrm{FC}\right)\:$MS (MT)/MS (G)	*P*-value	Significance	Trend MS (MT)/MS (G)	Related metabolic pathway
Hydroxybenzoic acid	1.3	7.3986	0.0061	**	Up	Phenylpropanoid biosynthesis
Serine	1.27	−5.3812	0.0059	**	Down	Glycine, serine and threonine metabolism
Dihydrosinapicacid	1.25	6.153	0.0079	**	Up	Phenylpropanoid biosynthesis
Isorhamnetin	1.22	4.8555	0.0064	**	Up	Flavonoid biosynthesis
Salicylic acid-O-hexoside	1.22	5.1081	0.0064	**	Up	Phenylpropanoid biosynthesis
LysoPC (16:0)	1.2	5.0626	0.0063	**	Up	Glycerophospholipid metabolism
Leucine	1.2	−4.0173	0.0064	**	Down	Valine, leucine and isoleucine degradation
Erucic acid	1.2	3.9918	0.0067	**	Up	Fatty acid biosynthesis
Syringetin-3-O-glucoside	1.2	3.8544	0.0070	**	Up	Flavonoid biosynthesis
Eicosenoic acid	1.18	3.8121	0.0066	**	Up	Biosynthesis of unsaturated fatty acids
Gluconapin	1.18	3.5335	0.0072	**	Up	Glucosinolate biosynthesis
Coumaric acid	1.17	3.0741	0.0076	**	Up	Phenylpropanoid biosynthesis
Hydroxy palmitic acid	1.16	2.9498	0.0079	**	Up	Fatty acid biosynthesis
Isorhapontigenin	1.16	2.5889	0.0061	**	Up	Stilbenoid, diarylheptanoid and gingerol biosynthesis
Glucotropaeolin	1.15	2.8889	0.0061	**	Up	Glucosinolate biosynthesis
Acetyl tyrosine	1.13	2.5905	0.0060	**	Up	Phenylalanine, tyrosine and tryptophan biosynthesis
Linoleoyl glycerol	1.12	2.1164	0.0094	**	Up	Glycerolipid metabolism
Ferulic acid	1.12	3.1022	0.0095	**	Up	Phenylpropanoid biosynthesis
Eicosapentaenoic acid	1.12	2.3508	0.0095	**	Up	Biosynthesis of unsaturated fatty acids
Syringic acid	1.1	3.8288	0.0098	**	Up	Phenylpropanoid biosynthesis
Hydroxy glucobrassicin	1.1	2.07651	0.0098	**	Up	Glucosinolate biosynthesis
Linolenoyl glycerol	1.1	3.7668	0.0099	**	Up	Glycerolipid metabolism
LysoPC 18:3	1.09	3.6504	0.0101	*	Up	Glycerophospholipid metabolism
Ellagic acid	1.08	2.5196	0.0109	*	Up	Phenylpropanoid biosynthesis
PG (12:0/16:0)	1.07	2.2925	0.0111	*	Up	Glycerophospholipid metabolism
Quercetin	1.06	3.2757	0.0110	*	Up	Flavonoid biosynthesis
Methyl Coumarin	1.05	3.2043	0.0110	*	Up	Flavonoid biosynthesis
Feruloyl tryptamine	1.05	3.1544	0.0113	*	Up	Tryptophan metabolism
Sinapic acid	1.04	2.8428	0.0123	*	Up	Phenylpropanoid biosynthesis
Hydroxy arachidonic acid	1.03	2.8	0.0125	*	Up	Biosynthesis of unsaturated fatty acids
Sinigrin	1.03	2.6	0.0127	*	Up	Glucosinolate biosynthesis
Pterostilbene	1.02	2.8427	0.0142	*	Up	Stilbenoid, diarylheptanoid and gingerol biosynthesis
Chlorogenic acid (3-O-Caffeoylquinic acid)	1.02	2.5	0.0160	*	Up	Phenylpropanoid biosynthesis
Phenylalanine	1.01	2.3	0.0152	*	Up	Phenylalanine, tyrosine and tryptophan biosynthesis
Kaempferol	1.01	2.07	0.0103	*	Up	Flavonoid biosynthesis
Hydroxy linolenic acid	1.01	2.03	0.0108	*	Up	Biosynthesis of unsaturated fatty acids

*VIP: Variable Importance in Projection, FC: fold change, MS (MT): MT-primed germinated mustard seeds, and MS (G): unprimed germinated mustard seeds (control).

*On a$\:{\mathrm{log}}_{2}\left(\mathrm{FC}\right)\:$scale, a fold change greater than 2 equals an actual fold change greater than 4, and less than 0.5 corresponds to a$\:{\mathrm{log}}_{2}\left(\mathrm{FC}\right)\:$value below − 1 (2^− 1^ = 0.5). Criteria requiring a$\:{\mathrm{log}}_{2}\left(\mathrm{FC}\right)\:$fold change greater than 2 or less than − 1 are very strict, filtering for massive metabolic shifts.

**Statistically significant at$\:\boldsymbol{P}<0.01$(High significance level).

*Statistically significant at$\:\boldsymbol{P}<0.05$(Standard significance level).

#### Amino acids

In the current study, the relative abundance of some amino acids, such as serine, leucine, and the dipeptide glycyl valine, decreased following dual treatments of MT-priming and sprouting (versus the unprimed sprouted group), suggesting that these compounds are consumed during processing to fuel various physiological functions required for seedling growth and antioxidant potential. Also, melatonin priming strongly activates downstream specialized metabolic pathways, as evidenced by the significant accumulation of key phenylpropanoid derivatives (phenolic acids and flavonoids) and glucosinolates (Fig. 1), consistent with previous studies^42–44^. Rather than accumulating as free pools, primary precursor amino acids are rapidly consumed and converted into specialized end-products as glucosinolates and flavonoids.

In contrast, some amino acids as phenylalanine and acetyl tyrosine along with feruloyl tryptamine were significantly upregulated following MT-priming and sprouting as melatonin helps break down stored reserves (like proteins) in the dormant seeds, making amino acids available for the germinating seedling. This observation came in line with a previous study which demonstrated that melatonin priming increased the level of essential amino acids, organic acids, and unsaturated fatty acids. improving the nutritional value of quinoa sprouts^45^.

These differential amino acids were significantly enriched in two metabolic pathways phenylalanine, tyrosine and tryptophan biosynthesis and valine, leucine and isoleucine degradation as presented in Fig. 4C.

#### Phenolic acids

Table 1 demonstrates significant shifts in phenolic acid content in mustard sprouts preliminarily primed with melatonin. Most notably, MT-primed sprouted samples exhibited a dramatic accumulation of phenolic acids specifically hydroxybenzoic, dihydrosinapic, coumaric, ferulic, syringic, and chlorogenic acids, along with salicylic acid-O-hexoxide reaching approximately 2.5 to 6 -fold increments compared to those found in unprimed sprouted samples, inferring that melatonin act as plant phytohormone which enzymatically triggers the biosynthesis and the release of bound phenolics. Parallel with a recent leading study, it was found that melatonin priming significantly increased phenolic acids content in barley seedlings through direct acting on the key enzymes in the phenylpropanoid pathway, such as phenylalanine ammonia-lyase (PAL) and cinnamic acid 4-hydroxylase (CAH), and ferulic acid-5-hydroxylase which are crucial for phenolic acid synthesis^46^.

As presented in Fig. 4C, the data demonstrated that phenylpropanoid biosynthesis is one of the primary metabolic pathways dramatically affected by MT-priming, closely correlating with significant fluctuations in total phenolic levels.

#### Glucosinolates

As presented in Table 1, it was notable that the relative abundance of glucosinolates witnessed 2 to 3-fold rise in mustard sprouts preliminarily exposed to MT-priming. More specifically, some aliphatic glucosinolates such as gluconapin, Glucotropaeolin, and sinigrin revealed a significant elevation with fold increments of 3.5, 2.9, and 2.6, respectively compared to unprimed controls, suggesting that melatonin acts as a potent signalling molecule that triggers biosynthetic pathways for these health-promoting secondary metabolites. Similarly, indolic glucosinolates as hydroxy glucobrassicin moderately increased (2-fold) in MT-primed mustard sprouts. A previous transcriptomic study demonstrated that melatonin treatment can upregulate key genes (such as CYP79F1 and CYP79B2) and transcription factors (like MYB28 and MYB34) involved in the glucosinolate biosynthetic pathway in some *Brassica* species (like broccoli)^47^.

As illustrated in the data (Fig. 4C), a prominent metabolic pathway that was noticeably altered in primed mustard sprouts was linked to glucosinolates biosynthesis.

#### Flavonoids and stilbenes

In the current work, flavonoids and their derivatives constitute a prominent class of putatively differential metabolites observed throughout the pairwise analysis. Coincidently, flavonoid biosynthesis was one of the top-ranked metabolic pathways significantly altered following MT-priming in mustard sprouts.

For clarity, a marked elevation in the relative content of some flavonoids primarily isorhamnetin, syringetin-O-glucoside, quercetin, methyl coumarin, and kaempferol in the primed germinated group, with fold changes ranging from 2 to 4 times that of the unprimed controls was observed, affirming the positive impact of melatonin application in augmenting the flavonoid content. In accordance with previous reports, it was found that seed priming with melatonin notably increases the antioxidants, flavonoid, and phenolic acid content, particularly under abiotic stresses such as salinity, drought, and chilling^19,20^. For instance, melatonin-primed tomato seeds exhibit significantly higher total flavonoid content (TFC) in seedlings grown under chilling stress (12–15 °C)^20^. In another study investigating the effect of melatonin priming of basil seeds (*Ocimum basilicum L.*) under salt stress, it was found that the interactive effects of melatonin and salt stress significantly favored the accumulation of total phenolics and flavonoids^48^. In the same context, some stilbenes such as isorhapontigenin and pterostilbene exhibited upregulating pattern in primed mustard sprouts compared to unprimed ones (Table 1).

Theses metabolic shifts are tightly connected with flavonoid biosynthesis as well as stilbenoid, diarylheptanoid and gingerol biosynthesis (Fig. 4C).

#### Fatty acids and their derivatives

In MT-primed mustard sprouts, fatty acids including saturated, unsaturated, and glyceride forms witnessed a moderate upward trend. As illustrated in Table 1, saturated fatty acids as erucic acid and hydroxy palmitic acid were noticed to be overexpressed by 3 and 2.5-fold in the primed sprouts compared to the others. Likewise, unsaturated fatty acids as eicosapentaenoic acid, eicosenoic acid, hydroxy arachidonic acid, and hydroxy linolenic acid along with some glycerides mainly, linoleoyl and linolenoyl glycerols presented a notable rise in the primed sprouted samples relative to the unprimed ones. This trend was consistent with a previously published study in safflower, which demonstrated that melatonin priming significantly boosted oil content and improved fatty acid profiles, especially under drought stress, signifying that melatonin priming helps seeds better utilize their reserves and produce high-quality nutritious oils^49^.

Essentially, following melatonin priming and sprouting of mustard seeds, the levels of lysophosphatidylcholines (LPCs), such as LysoPC (16:0) and LysoPC (18:3), along with PG (12:0/16:0), notably increased compared to unprimed controls. This rise can be attributed to the active metabolism of stored lipids and the remodeling of cell membranes to generate energy and build new tissues^50^. This pattern in phospholipid composition aligned with a previous report on foxtail millet seedlings under drought stress^50^.

KEGG enrichment analysis (Fig. 4C) demonstrated that these lipid shifts in MT-primed mustard sprouts were primarily associated with the biosynthesis of unsaturated fatty acids and glycerophospholipid metabolism pathways.

In summary, this study represents the first attempt to map key differential metabolites to their respective metabolic pathways, uncovering the chemical patterns and regulatory metabolic pathway networks in MT-primed mustard sprouts.

### Acetylcholine esterase and monoamine oxidase inhibitory activities of different mustard samples

With a scope to unearth the dual promising effect of MT-priming and sprouting in remodelling the chemical profile and biological traits of mustard seeds, raw seeds, unprimed, and MT-primed mustard sprouts were in vitro screened against AChE and MAO-B enzymes which serve as remarkable pharmacological targets for AD to supplement the prior metabolic results, unravelling a clearer connection between the accumulation of specific bioactive compounds and the MT-primed sprouted samples’ potential health benefits against AD.

As presented in Table 2, in vitro AChE and MAO-B inhibitory assays revealed that all mustard extracts demonstrated significant, dose-dependent inhibitory effects on both enzymes. The most noteworthy observations were recorded for the MT-primed sprouted samples, which exhibited respective IC_50_ values equal 7.667 ± 0.306 mg/dl and 45.389 ± 0.953 compared to the unprimed controls (IC_50_ = 20.865 ± 0.626 mg/dl and 82.646 ± 2.042 mg/dl). Of note, the raw ungerminated seeds showed the weakest inhibitory activity against AChE and MAO-B, with IC_50_ values of 39.995 ± 1.053 mg/dl and 90.896 ± 1.729 mg/dl, respectively, highlighting the positive impact of germination and priming in enhancing the biological traits of the raw seeds. The dose-response curves of all tested samples and the positive controls (Physostigmine and Pargyline) are clarified under supplementary material. Worthily, our findings suggested that melatonin priming acts as a robust biostimulator, reprogramming seed metabolism and significantly boosting the biological attributes.

**Table 2:** In vitro AChE and MAO-B inhibitory activity results of raw seeds, unprimed, and MT-primed mustard sprouts.

Mustard sample	Acetylcholinesterase (AChE) IC_50_ (mg/dl)	Monoamine oxidase (MAO-B) IC_50_ (mg/dl)
MT-primed germinated mustard seeds (MS (MT))	7.667 ± 0.306^a^	45.389 ± 0.953^e^
Unprimed germinated mustard seeds (MS (G))	20.865 ± 0.626^b^	82.646 ± 2.042^f^
Raw seeds	39.995 ± 1.053^c^	90.896 ± 1.729^f^
Physostigmine (positive control)	6.578 ± 0.089^a^	……….
Pargyline (positive control)	……….	52.606 ± 0.472^e^
F	**1982**	**746**
P	**< 0.001** ^*^	**< 0.001** ^*^

F: F for One way ANOVA test, pairwise comparison bet. each of the 2 groups).

p: p value for comparing between the studied groups.

Any Common letter ^(a−f)^ are not significant (**or** means with totally Different letters ^(a−f)^ are significant).

Statistical analysis and one-way ANOVA with Tukey’s post-hoc pairwise comparisons were performed using Graph Pad InStat Software Inc.,.

Program, version 8.1.0 Philadelphia, San Diego CA, USA.

### OPLS directed analysis for unveiling the efficacy compounds beyond acetylcholine esterase and monoamine oxidase inhibitory activities in the different mustard samples

To enhance the biological interpretability of the findings, an Orthogonal Projection to Latent Structures (OPLS) predictive model was employed to identify potential bioactive metabolites that collectively mediate AChE and MAO-B inhibitory activities.

**Fig. 5:**
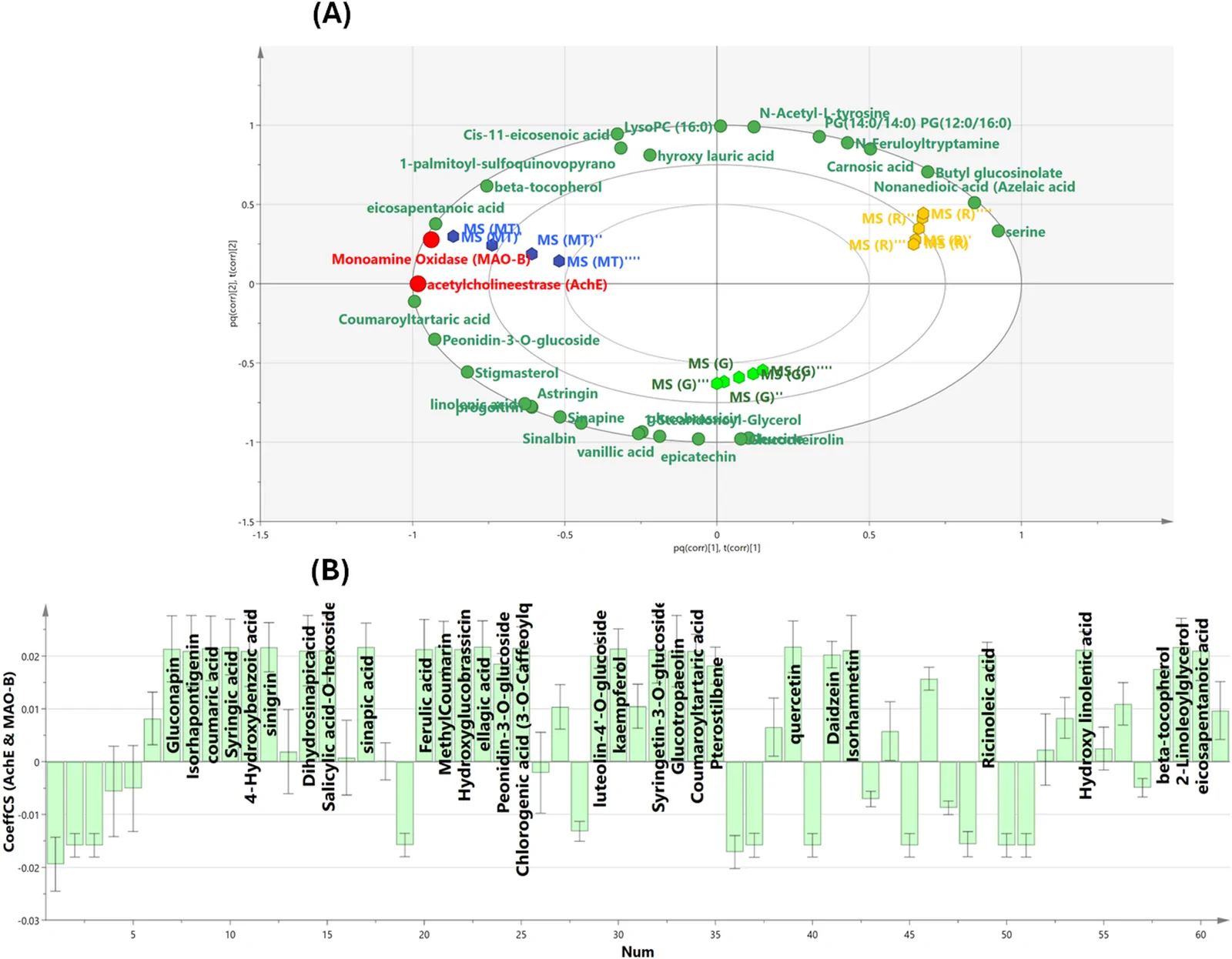
**(A)** OPLS biplot distinguishing between raw, germinated (unprimed), and MT-primed germinated mustard samples based on their AChE and MAO-B inhibitory activities, **(B)** OPLS-derived coefficients plot pinpointing the efficacy compounds in in MT-primed germinated samples that possibly underlie their notable AChE and MAO-B inhibitory activities.

OPLS biplot (Fig. 5A) accounting for 70.3% of the total variance effectively distinguished between raw, germinated (unprimed), and MT-primed germinated mustard samples based on their AChE and MAO-B inhibitory potentials, where MT-primed mustard sprout samples clustered along the positive axis of latent variable 1 (LV1), showing a spatial correlation with AChE and MAO-B inhibitory activities (Y-variables), while raw samples were positioned distantly from these variables, suggesting less inhibition against AChE and MAO-B enzymes. Similarly, unprimed samples were allocated roughly away from AChE and MAO-B inhibitory activities (Y-variables), aligning with the prior biological findings. R_2_Y and Q^2^, respectively calculated as 0.973 and 0.968 affirmed the predictive reliability of the OPLS model.

The OPLS-derived coefficient plot was further integrated to probe into the possible efficacy compounds beyond AChE and MAO-B inhibitory activities of MT-primed sprouted samples. Analysis of the OPLS-derived coefficients plot (Fig. 5B) indicated that the significant AChE and MAO-B inhibitory activities observed in MT-primed germinated samples is might be statistically correlated with their high concentrations of bioactive phenolic acids, primarily syringic, coumaric, ferulic, ellagic, and chlorogenic acids along with some flavonoids and stilbenes such as syringetin-3-O-glucoside, kaempferol, quercetin, and pterostilbene. Additionally, some major glucosinolates, including sinigrin, gluconapin, hydroxy glucobrassicin, and glucotropaeolin may be primarily responsible for the significant AChE and MAO-B inhibitory activities observed in primed mustard sprouts. Some fatty acids as eicosapentaenoic acid, ricinoleic acid, and hydroxy linolenic acid as well as beta-tocopherol might be responsible for these enhanced inhibitory effects based on multivariate statistical correlation. Worth noting, these observations came in line with previous studies which delineated the multi-target neuroprotective potential of phenolic acids through inhibitory acting on AChE and MAO-B enzymes critical in the progression of Alzheimer’s disease^51,52^. For instance, a previous report has demonstrated that ellagic acid acts as a dual-targeting competitive inhibitor for both AChE and MAO-B enzymes with IC_50_ values equal 41.7 µM and 9.21 µM, respectively^52^. Another in vivo study revealed the neuroprotective effect and anti-acetylcholinesterase activity of chlorogenic acid in scopolamine-induced amnesia mouse model, outlining that chlorogenic acid might exhibit anti-amnesic effect via dose-dependent inhibition of AChE activity and malondialdehyde in the hippocampus and frontal cortex^53^. Equally important, a recent leading investigation has proved the neuroprotective effect of syringic acid in rat model of Traumatic Brain Injury (TBI) through attenuating increased AChE activity and restoring antioxidant levels^54^. On other domains, in accordance with previous investigations, quercetin, kaempferol, and pterostilbene exhibit both AChE and MAO-B inhibitory activities, making them potential dual-acting inhibitors for neurodegenerative conditions like AD^55–57^. Eicosapentaenoic acid is an omega-3 fatty acid well-recognized for its neuroprotective effects and role in cognitive function^58^.

While this study provides a foundational biochemical framework for melatonin-mediated biostimulation in brown mustard, several limitations highlight important avenues for future investigation. First, while quantitative metabolomics and KEGG pathway enrichment clearly identified marked metabolic shifts, direct transcriptomic profiling (RT-qPCR of candidate genes such as *PAL*, *CYP79F1*, and *MYB28*) and targeted enzymatic activity assays remain necessary to explicitly confirm the underlying transcriptional and post-translational regulatory mechanisms. Second, because this work evaluated a single cultivar under controlled germination conditions to minimize confounding genotype-by-environment variations, extending this priming protocol across diverse *Brassica* genotypes and related species such as broccoli and kale, as well as validating efficacy under multi-stress field conditions, will be critical to demonstrate its broader agronomic applicability and genotype-specific responses. Third, our findings are limited by their reliance on multivariate statistical correlations rather than the independent testing of pure bioactive compounds to establish direct causality, highlighting the need for future biological validation using isolated pure compounds to confirm their individual inhibitory potencies.

## Conclusion

Inspired by the nutritional and therapeutic attributes of mustard seeds rooted in traditional medicine, this study presents the first attempt to monitor the chemical shifts and biological traits observed in mustard seeds post-exposure to MT-priming and subsequent sprouting. Given this, MT-primed mustard sprouts exhibited a remarkable elevation in phenolic acids, flavonoids, stilbenes, glucosinolates, and some unsaturated fatty acids compared to unprimed sprouted ones. These metabolic shifts were primarily associated with phenylpropanoid biosynthesis, flavonoid biosynthesis, glucosinolate biosynthesis and stilbenoid, diarylheptanoid and gingerol biosynthesis, and the biosynthesis of unsaturated fatty acids. Regarding AChE and MAO-B inhibitory screening, the MT-primed sprouted samples recorded the most noteworthy observations, with respective IC_50_ values equal 7.667 ± 0.306 mg/dl and 45.389 ± 0.953 compared to unprimed controls (IC_50_ = 20.865 ± 0.626 mg/dl and 82.646 ± 2.042 mg/dl). OPLS correlation analysis portrayed that MT-primed germinated samples were enriched with various biologically active compounds, primarily syringic, ellagic, and chlorogenic acids, along with quercetin, pterostilbene, sinigrin, gluconapin, and eicosapentaenoic acid, which may have underpinned their notable AChE and MAO-B inhibitory activities.

Overall, our findings indicated that melatonin priming acted as a robust biostimulator, reprogramming seed metabolism, tuning the chemical pattern, and significantly boosting biological attributes. However, future integrated analyses combining transcriptomics and metabolomics should be designed to comprehensively understand the core regulatory genes governing metabolic pathways, chemical patterns, and biological attributes.

## Supplementary Information

Below is the link to the electronic supplementary material.

Supplementary Material 1

## Data Availability

All data generated or analysed during this study are included in this published article [and its supplementary information files].

## References

[1] Cummings, J. Alzheimer’s disease diagnostic criteria: Practical applications. *Alzheimers Res. Ther.* **4**35. (2012). 22947665 10.1186/alzrt138 PMC3580392

[2] Ghallab, D. S., Awwad, Z. M., Ibrahim, R. S. & Shawky, E. Dietary relevance of sage (*Salvia officinalis* L.) in cognitive health: A serum-based mechanistic study targeting Alzheimer’s disease pathways. *Food Biosci.* **69**, 106863 (2025).

[3] Zhang, X. X. et al. The epidemiology of Alzheimer’s disease modifiable risk factors and prevention. *J. Prev. Alzheimer’s Dis.* **8**, 313–321 (2021). 34101789 10.14283/jpad.2021.15 PMC12280729

[4] Moreta, M.-G., Burgos-Alonso, N., Torrecilla, M., Marco-Contelles, J. & Bruzos-Cidón, C. Efficacy of acetylcholinesterase inhibitors on cognitive function in Alzheimer’s disease. Review of reviews. *Biomedicines* **9**, 1689 (2021). 34829917 10.3390/biomedicines9111689 PMC8615650

[5] Ruangritchankul, S., Chantharit, P., Srisuma, S. & Gray, L. C. Adverse drug reactions of acetylcholinesterase inhibitors in older people living with dementia: A comprehensive literature review. *Ther. Clin. Risk Manag.* 10.2147/TCRM.S323387 (2021). 34511919 PMC8427072

[6] Szőllősi, R. Indian mustard (Brassica juncea L.) seeds in health. In *Nuts and seeds in health and disease prevention* 357–364 (Elsevier, 2020).

[7] Rahman, M., Khatun, A., Liu, L. & Barkla, B. J. Brassicaceae mustards: Phytochemical constituents, pharmacological effects, and mechanisms of action against human disease. *Int. J. Mol. Sci.* **25**, 9039 (2024). 39201724 10.3390/ijms25169039 PMC11354652

[8] Grygier, A. Mustard seeds as a bioactive component of food. *Food Rev. Int.* **39**, 4088–4101 (2023).

[9] Goetz, K., Hinz, A., Steinhäuser, J. & Von Rath, U. Use of mustard seed footbaths for respiratory tract infections: A pilot study. *Evidence-Based Complement. Altern. Med.* **2020**, 5648560 (2020). 10.1155/2020/5648560 PMC7001671 32047526

[10] Das, G. et al. Glucosinolates and Omega-3 fatty acids from mustard seeds: Phytochemistry and pharmacology. *Plants* **11**, 2290 (2022). 36079672 10.3390/plants11172290 PMC9459965

[11] Akhtar, M. J. & Khan, S. A. Chemistry and biological activity of mustard oil: Therapeutic benefits and risk to healthcare. *Rev. Bras. Farmacogn.* **34**, 65–79 (2024).

[12] Ghallab, D. S., Ghareeb, D. A. & Goda, D. A. Integrative metabolomics and chemometrics depict the metabolic alterations of differently processed red kidney beans (*Phaseolus vulgaris* L.) and in relation to in-vitro anti-diabetic efficacy. *Food Res. Int.* 10.1016/j.foodres.2024.114786 (2024). 39147477

[13] Li, M. et al. Effects of processing method on chemical compositions and nutritional quality of ready-to‐eat sea cucumber (*Apostichopus japonicus*). *Food Sci. Nutr.* **7**, 755–763 (2019). 30847154 10.1002/fsn3.921 PMC6393041

[14] Gunathunga, C. et al. Germination effects on nutritional quality: A comprehensive review of selected cereals and pulses changes. *J. Food Compos. Anal.* **128**, 106024 (2024).

[15] Fatemi, H. Effect of bio-priming with plant growth promoting bacteria on growth and biochemical characteristics, phenol, flavonoid, vitamin C and nitrate in lettuce (*Lactuca sativa* L.) rabicon cultivar in different growth substrates. *J. Soil. Plant. Interact. Univ. Technol.* **11**, 41–59 (2020).

[16] Mondal, S. & Bose, B. Impact of micronutrient seed priming on germination, growth, development, nutritional status and yield aspects of plants. *J. Plant. Nutr.* **42**, 2577–2599 (2019).

[17] Ghallab, D. S., Ghareeb, D. A., Shawer, E. E., Othman, A. A. & Elattar, M. M. Microbial biopriming and germination cooperatively remodel brown lentil seeds (*Lens culinaris* L.) metabolome and antidiabetic functionality. *npj Sci. Food* **10**, 33 (2026). 41974744 10.1038/s41538-026-00824-5 PMC13194844

[18] González-García, Y. et al. Seed priming with carbon nanomaterials improves the bioactive compounds of tomato plants under saline stress. *Plants* **11**, 1984 (2022). 35956461 10.3390/plants11151984 PMC9370608

[19] Rajora, N. et al. Seed priming with melatonin: A promising approach to combat abiotic stress in plants. *Plant Stress* **4**, 100071 (2022).

[20] Park, K. et al. Seed priming with melatonin enhances chilling stress tolerance in tomato seedlings. *Hortic. Plant J.* 10.1016/j.hpj.2025.02.017 (2025).

[21] Chen, C. et al. Facilitating maize seed germination under heat stress via exogenous melatonin. *Int. J. Mol. Sci.* **26**, 1608 (2025). 40004072 10.3390/ijms26041608 PMC11855634

[22] Askari, M. et al. Exogenous melatonin application stimulates growth, photosynthetic pigments and antioxidant potential of white beans under salinity stress. *S. Afr. J. Bot.* **160**, 219–228 (2023).

[23] Ahmad, K. et al. Melatonin seed priming enhanced drought tolerance in rapeseed (*Brassica napus* L.) cultivars. *Sci. Rep.* 10.1038/s41598-026-56134-z (2026). 42230725 PMC13392096

[24] Ali, M., Pan, Y., Liu, H. & Cheng, Z. Melatonin interaction with abscisic acid in the regulation of abiotic stress in *Solanaceae* family plants. *Front. Plant Sci.* **14**, 1271137 (2023). 37767290 10.3389/fpls.2023.1271137 PMC10520282

[25] Ghallab, D. S., Mabrouk, M. E. M. & Naga, N. G. Comparison of metabolic profiles, regulatory pathways, and quorum sensing inhibition activity of brown mustard seeds (*Brassica juncea* L.) post germination and roasting treatments. *Npj Sci. Food* **9**221. (2025). 41219224 10.1038/s41538-025-00606-5 PMC12606101

[26] Yu, Y., Deng, L., Zhou, L., Chen, G. & Wang, Y. Exogenous melatonin activates antioxidant systems to increase the ability of rice seeds to germinate under high temperature conditions. *Plants* **11**, 886 (2022). 35406866 10.3390/plants11070886 PMC9003151

[27] Awan, S. A. et al. Pre-treatment of melatonin enhances the seed germination responses and physiological mechanisms of soybean (*Glycine max* L.) under abiotic stresses. *Front. Plant Sci.* **14**, 1149873 (2023). 36950358 10.3389/fpls.2023.1149873 PMC10025545

[28] Ghallab, D. S. & Ghareeb, D. A. UPLC-MS/MS-based metabolomics and chemometrics unveil metabolic patterns and their regulatory pathways over the course of pumpkin seeds (*Cucurbita maxima*) germination. *Sci. Hortic. (Amsterdam)* **337**, 113563 (2024).

[29] Ghallab, D. S., Ibrahim, R. S., Ghareeb, D. A. & El Newehy, N. M. Multi-omics for unveiling potential antidiabetic markers from red, green and black mung beans using NIR-UPLC-MS/MS multiplex approach. *Sci. Rep.* 10.1038/s41598-025-03911-x (2025). 40451910 PMC12127463

[30] El-Banna, A. A., Ibrahim, R. S. & Ghallab, D. S. Unlocking hidden biomarkers: Exploring miswak (*Salvadora persica* L.) potential in tongue squamous cell carcinoma through UPLC-MS/MS and multivariate analysis. *South. Afr. J. Bot.* **180**, 497–511 (2025).

[31] Ghallab, D. S., Nasr, S. A., Ghareeb, D. A., Abd, E. & Hafez, M. S. M. Cooking and salting impacts on sea cucumber (*Holothuria atra*) metabolome and their entanglements on anticoagulant potential as revealed by UPLC-QqQ-MS/MS analysis and chemometrics. *J Food Compos. Anal* 106911 (2024).

[32] Singh, D. P. et al. Untargeted metabolomics of Alternaria solani-challenged wild tomato species *Solanum cheesmaniae* revealed key metabolite biomarkers and insight into altered metabolic pathways. *Metabolites* **13**, 585 (2023). 37233626 10.3390/metabo13050585 PMC10220610

[33] Ghallab, D. S. et al. Unveiling the pharmacological mechanisms of Spirulina platensis in rheumatoid arthritis rats through the integration of serum metabolomics, pathways analysis, and experimental validation. *Naunyn Schmiedebergs Arch. Pharmacol* 1–19 (2025). 10.1007/s00210-025-04191-y PMC12552235 40332553

[34] Kanehisa, M., Furumichi, M., Sato, Y., Matsuura, Y. & Ishiguro-Watanabe, M. KEGG: biological systems database as a model of the real world. *Nucleic Acids Res.* **53**, D672–D677 (2025). 39417505 10.1093/nar/gkae909 PMC11701520

[35] Kanehisa, M. & Goto, S. KEGG: kyoto encyclopedia of genes and genomes. *Nucleic Acids Res.* **28**, 27–30 (2000). 10592173 10.1093/nar/28.1.27 PMC102409

[36] Abou-Taleb, B. A. et al. Comparison of *Peganum harmala* L. leaves extract nanoformulations against herpes simplex virus type 1 guided by network pharmacology analysis. *Sci. Rep.* **15**, 40395 (2025). 41253903 10.1038/s41598-025-24155-9 PMC12627805

[37] Ghallab, D. S., Ghareeb, D. A., Sherif, M. & Kenawy, M. Y. Systematic evaluation of a novel mesoporous silica nanoparticles (MSNs)-*Spirulina platensis* nanohybrid for breast cancer treatment: Mechanistic investigation via pharmacological network analysis. *RSC Adv.* **16**, 4347–4362 (2026). 41568217 10.1039/d5ra08876c PMC12817816

[38] Ellman, G. L., Courtney, K. D., Andres, V. Jr. & Featherstone, R. M. A new and rapid colorimetric determination of acetylcholinesterase activity. *Biochem. Pharmacol.* **7**, 88–95 (1961). 13726518 10.1016/0006-2952(61)90145-9

[39] Youdim, M. B. H. & Bakhle, Y. S. Monoamine oxidase: isoforms and inhibitors in Parkinson’s disease and depressive illness. *Br. J. Pharmacol.* **147**, S287–S296 (2006). 16402116 10.1038/sj.bjp.0706464 PMC1760741

[40] Magnotti, R. A. Jr., Eberly, J. P., Quarm, D. E. & McConnell, R. S. Measurement of acetylcholinesterase in erythrocytes in the field. *Clin. Chem.* **33**, 1731–1735 (1987). 3665026

[41] Binda, C., Newton-Vinson, P., Hubálek, F., Edmondson, D. E. & Mattevi, A. Structure of human monoamine oxidase B, a drug target for the treatment of neurological disorders. *Nat. Struct. Biol.* **9**, 22–26 (2002). 11753429 10.1038/nsb732

[42] Xu, Y. et al. Metabolite and transcriptome profiling analysis provides new insights into the distinctive effects of exogenous melatonin on flavonoids biosynthesis in *Rosa rugosa*. *Int. J. Mol. Sci.* **25**, 9248 (2024). 39273197 10.3390/ijms25179248 PMC11395435

[43] Wei, L., Liu, C., Zheng, H. & Zheng, L. Melatonin treatment affects the glucoraphanin-sulforaphane system in postharvest fresh-cut broccoli (*Brassica oleracea* L.). *Food Chem.* **307**, 125562 (2020). 31648174 10.1016/j.foodchem.2019.125562

[44] Gao, F., Han, K., Ma, W., Zhang, J. & Xie, J. Exogenous melatonin application enhances pepper (*Capsicum annuum* L.) fruit quality via activation of the phenylpropanoid metabolism. *Foods* **14**, 1247 (2025). 40238541 10.3390/foods14071247 PMC11988627

[45] Zrig, A. et al. Melatonin priming as a promising approach to improve biomass accumulation and the nutritional values of Chenopodium quinoa sprouts: A genotype-based study. *Sci. Hortic. (Amsterdam)*. **301**, 111088 (2022).

[46] Tian, X. et al. Melatonin mediates phenolic acids accumulation in barley sprouts under MeJA stress. *Front. Nutr.* **11**, 1403293 (2024). 38899320 10.3389/fnut.2024.1403293 PMC11186395

[47] Tian, P. et al. Transcriptomics analysis of genes induced by melatonin related to glucosinolates synthesis in broccoli hairy roots. *Plant Signal. Behav.* **16**, 1952742 (2021). 34545770 10.1080/15592324.2021.1952742 PMC8526036

[48] Bahcesular, B., Yildirim, E. D., Karaçocuk, M., Kulak, M. & Karaman, S. Seed priming with melatonin effects on growth, essential oil compounds and antioxidant activity of basil (*Ocimum basilicum* L.) under salinity stress. *Ind. Crops Prod.* **146**, 112165 (2020).

[49] Akbari, G. A., Heshmati, S., Soltani, E. & Amini Dehaghi, M. Influence of seed priming on seed yield, oil content and fatty acid composition of safflower (*Carthamus tinctorius* L.) grown under water deficit. *Int. J. Plant Prod.* **14**, 245–258 (2020).

[50] Ren, J. et al. Melatonin improves drought stress tolerance by remodeling lipid metabolism in *Setaria italica* L. *Plants* **14**, 3314 (2025). 41225864 10.3390/plants14213314 PMC12608707

[51] Caruso, G. et al. Phenolic acids and prevention of cognitive decline: Polyphenols with a neuroprotective role in cognitive disorders and Alzheimer’s disease. *Nutrients* **14**, 819 (2022). 35215469 10.3390/nu14040819 PMC8875888

[52] Oh, J. M. et al. Acetylcholinesterase and monoamine oxidase-B inhibitory activities by ellagic acid derivatives isolated from *Castanopsis cuspidata* var. *sieboldii*. *Sci. Rep.* **11**13953. (2021). 34230570 10.1038/s41598-021-93458-4 PMC8260592

[53] Kwon, S.-H. et al. Neuroprotective effects of chlorogenic acid on scopolamine-induced amnesia via anti-acetylcholinesterase and anti-oxidative activities in mice. *Eur. J. Pharmacol.* **649**, 210–217 (2010). 20854806 10.1016/j.ejphar.2010.09.001

[54] Banderwal, R. & Kumar, A. Neuroprotective mechanism of syringic acid targeting oxidative damage and neuroinflammation in an experimental model of traumatic brain injury. *Inflammopharmacology* 1–18 (2025). 10.1007/s10787-025-02075-4 41369867

[55] Grewal, A. K. et al. Mechanistic insights and perspectives involved in neuroprotective action of quercetin. *Biomed. Pharmacother.* **140**, 111729 (2021). 34044274 10.1016/j.biopha.2021.111729

[56] Jin, S., Zhang, L. & Wang, L. Kaempferol, a potential neuroprotective agent in neurodegenerative diseases: From chemistry to medicine. *Biomed. Pharmacother.* **165**, 115215 (2023). 37494786 10.1016/j.biopha.2023.115215

[57] Gao, S. et al. Pterostilbene: A natural neuroprotective stilbene with anti-Alzheimer’s disease properties. *J. Pharm. Anal.* **15**, 101043 (2025). 40291020 10.1016/j.jpha.2024.101043 PMC12032911

[58] Dighriri, I. M. et al. Effects of omega-3 polyunsaturated fatty acids on brain functions: A systematic review. *Cureus*10.7759/cureus.30091 (2022). 36381743 PMC9641984

