# Supplementary material for: Melatonin seed priming reconfigures the metabolomic profile and functional attributes of brown mustard (Brassica juncea L.) sprouts
Source: Sci Rep. 2026 Sep 3;16:27654. doi: 10.1038/s41598-026-68267-2 (PMC13542083; doi:10.1038/s41598-026-68267-2)
Supplement: Supplementary file 1 [file 41598_2026_68267_MOESM1_ESM.docx]

*Phytochemical analysis of different mustard extracts using UPLC-MS/MS*

Metabolic proﬁling of different mustard extracts was performed on a Shimadzu 8045 UPLC triple quadruple (QqQ) apparatus (Shimadzu corporation, Kyoto, Japan) coupled with an electrospray ionization (ESI) source. The apparatus is equipped with a pump (LC 2040), an autosampler (SIL-30AC), a detector (LC-2030/2040 PDA detector), and an on-line degasser. Chromatographic separation was conducted using a Shimpack GISS UPLC C18 column (50 mm × 2.1 mm ID × 1.7 μm particle size) (Shimadzu corporation, Kyoto, Japan) maintained at 30°C. The biphasic mobile phase consisted of acidiﬁed ultrapure water ( 0.1% formic acid) (Phase A) and acetonitrile (Phase B) was gradient eluted at a ﬂow rate of 0.2 mL /min and programmed as follows: 5% B at retention time of 0.0–10.0 min, 40% B at 10.0–14.0 min, 50% B at 14.0–18.0 min, 70% B at 18.0–22.0 min, 90% B at 22.0–26.0 min, 95% at 26.0–30.0 min, 100% B at 30.0–35.0 min and ﬁnally returning to the initial condition at 35 min. The suitably chosen set of operational parameters for ESI interface operated in both positive and negative ion modes with full scan function covering the mass range of 50–1200 Da were set according to our previous publication ^1^ as follows: capillary voltage of 3 kV, cone voltage; 35 V, the ion source temperature was 150◦C, the nebulizer (nitrogen gas) pressure was 35 psi, drying and sheath gas (N_2_) temperature was 440 ^◦^C and 350 ^◦^C, respectively. The drying and sheath gas ﬂows were applied at 900 L/h and 50 L/h, respectively. The scan time and interscan delay were set to 0.4 s and 0.1 s, respectively and the analytical run time was extended to 35 min. Tandem mass spectrometry (MS/MS) fragmentation was acquired in Product Ion Scan mode using high-purity argon as the collision gas in the Q2 collision cell, with collision energies (CE) optimized between $10\text{ eV}$ and $45\text{ eV}$ to obtain characteristic precursor-to-product ion spectrum profiles.

The data processing was conducted using MZmine 2.0 analysis software. The processing parameters were set as follows: mass range 100–1000 Da, mass tolerance 0.15 Da, RT tolerance (min) 0.2, signal-to-noise threshold 1.5. Afterwards, a data matrix compressing the retention time, mass-to-charge ratio (m/z) and normalized peak area was generated. Metabolite annotations were performed in compliance with the Metabolomics Standards Initiative (MSI) criteria. Level 1 confirmation was established for metabolites (including sinigrin, quercetin, ferulic acid, stigmasterol, and leucine) by comparing retention times, precursor $m/z$, and MS/MS spectrum fragments directly against authentic external commercial standards. Level 2 identification was assigned using our in-house database, tandem MS spectral matching, relevant literature, and the Dictionary of Natural Products (DNP) database. Of note, 5 µl from each sample were pooled to create quality control (QC) samples for assessing the reproducibility and robustness of the analytical platform.

**Table S1: List of identified compounds detected in the raw seed, unprimed and MT-primed mustard sprout extracts through UPLC-MS/MS analysis**

| **No.** | **Rt (min.)** | **Identified compounds** | **Precursor ions** | **Chemical formulas** | **Main fragments (Da)** | **Chemical classes** | **HMDB ID** | **References** | **Source (Presence)** |
| --- | --- | --- | --- | --- | --- | --- | --- | --- | --- |
| 1 | 0.76 | Allyl isothiocyanate | 100.2  M+H | [C_4_H_5_NS](https://pubchem.ncbi.nlm.nih.gov/#query=C4H5NS) | 72 | Isothiocyanates | HMDB0005843 | ^2^ | Raw seeds, Unprimed mustard sprouts, MT-primed mustard sprouts |
| 2 | 0.87 | Gamma amino butyric acid (GABA) | 104.2  M+H | C_4_H_9_NO_2_ | 87-60 | Amino acids | HMDB0000112 | ^3^ | Raw seeds, Unprimed mustard sprouts, MT-primed mustard sprouts |
| 3 | 0.9 | Serine | 106.2  M+H | C_3_H_7_NO_3_ | 88-71 | Amino acids | HMDB0000187 | ^4^ | Raw seeds, Unprimed mustard sprouts, MT-primed mustard sprouts |
| 4 | 1.12 | Sinigrin* | 358.04  M-H | C_10_H_17_NO_9_S_2_ | 274-195-96 | Allyl glucosinolates | HMDB0034070 | …… | Raw seeds, Unprimed mustard sprouts, MT-primed mustard sprouts |
| 5 | 1.2 | Glycyl valine | 175.1  M+H | C_7_H_14_N_2_O_3_ | 101-76 | Dipeptides | HMDB0028854 | ^5^ | Raw seeds |
| 6 | 1.3 | Glutamyl leucine | 260.3  M+H | C_11_H_20_N_2_O_5_ | 132-103 | Dipeptides | HMDB0011171 | ^6^ | Raw seeds, Unprimed mustard sprouts, MT-primed mustard sprouts |
| 7 | 1.42 | Leucine* | 132.2  M+H | C_6_H_13_NO_2_ | 103-86 | Amino acids | HMDB0013773 | ……. | Raw seeds, Unprimed mustard sprouts, MT-primed mustard sprouts |
| 8 | 1.5 | Glucocheirolin | 438.4  M-H | C_11_H_21_NO_11_S_3_ | 332-259-195-135-96-74 | Aliphatic glucosinolates | HMDB0000101 | ^7^ | Unprimed mustard sprouts, MT-primed mustard sprouts |
| 9 | 1.6 | [Progoitrin](https://pubchem.ncbi.nlm.nih.gov/compound/Epi-progoitrin) | 388.3  M-H | C_11_H_19_NO_10_S_2_ | 332-259-195-96-74 | Aliphatic glucosinolates | HMDB0034071 | ^8^ | Unprimed mustard sprouts, MT-primed mustard sprouts |
| 10 | 1.7 | Gluconapin | 372.3  M-H | [C_11_H_18_NO_9_S_2_^-^](https://pubchem.ncbi.nlm.nih.gov/#query=C11H18NO9S2-) | 274-259- 195-96-74 | Aliphatic glucosinolates | HMDB0000426 | ^8^ | Unprimed mustard sprouts, MT-primed mustard sprouts |
| 11 | 1.8 | Butyl glucosinolate | 374.2  M-H | [C_11_H_21_NO_9_S_2_](https://pubchem.ncbi.nlm.nih.gov/#query=C11H21NO9S2) | 96-95-74 | Aliphatic glucosinolates | HMDB0038403 | ^9^ | MT-primed mustard sprouts |
| 12 | 2.4 | Indole 3-acetic acid (IAA) | 176.2  M+H | C₁₀H₉NO₂ | 130-103 | Phytohormones ([auxins](https://www.google.com/search?q=auxins&sca_esv=1c5310715e652d88&sxsrf=AE3TifPIMYYvMnuxA28J3oVrmYV2rQhf9w%3A1767312803546&ei=ow1XabSSIbyLkdUPx9rlkA4&ved=2ahUKEwjy7NC-yeuRAxWuSaQEHdQCN3gQgK4QegQIAhAC&uact=5&oq=Indole+3-acetic+acid+%28IAA%29+chemical+class&gs_lp=Egxnd3Mtd2l6LXNlcnAiKUluZG9sZSAzLWFjZXRpYyBhY2lkIChJQUEpIGNoZW1pY2FsIGNsYXNzMgUQIRigAUjCQVDaBFi5PHACeACQAQCYAZwBoAGEEKoBBDAuMTi4AQPIAQD4AQGYAhOgApUQwgIHECMYsAMYJ8ICChAAGLADGNYEGEfCAgoQIxjwBRgnGJ4GwgIEECMYJ8ICCBAAGIAEGKIEwgIFEAAY7wXCAgYQABgWGB7CAggQABgWGAoYHsICCxAAGIAEGIYDGIoFwgIHECEYoAEYCsICBBAhGBWYAwCIBgGQBgmSBwQyLjE3oAfyYbIHBDAuMTe4B4QQwgcGMC4xMC45yAdDgAgA&sclient=gws-wiz-serp&mstk=AUtExfCmt9Hp8Q6VQ9848SFoHl7cXB5ZQSBt8J12fyqSMMVckhx3aev0Kb4LUeSHaAWw-iDOGmWjGpA65TvTydGEf_JzRrP7LU2qIdysZVSMy-_FvbLmK9phRUjGoujD2l4mOow3VdJaCzBbodN5qkcMc1oGTTAVRaiZFwcTFsvWuXEiYZmjeUs2io6bTFidQHfrWwd1heFDWItnA8qphw582xiqIwwYFiD46CX9oLTQn2539tnfjkT8mvP1hk5zD4Mbef4MoFtfYFt9dZXAwPB0-qWM&csui=3)) | HMDB0000197 | ^10^ | Raw seeds, Unprimed mustard sprouts, MT-primed mustard sprouts |
| 13 | 2.5 | Glucotropaeolin | 408.3  M-H | C_14_H_19_NO_9_S_2_ | 259-195-166-96-74 | Aromatic glucosinolates | HMDB0038419 | ^9^ | Unprimed mustard sprouts, MT-primed mustard sprouts |
| 14 | 2.6 | Gluconasturtiin | 421.3  M-H | C_15_H_21_NO_9_S_2_ | 274-259- 195-96-74 | Aromatic glucosinolates | HMDB0038423 | ^11^ | Unprimed mustard sprouts, MT-primed mustard sprouts |
| 15 | 2.75 | Hydroxyglucobrassicin | 463.2  M-H | C_16_H_20_N_2_O_10_S_2_ | 447-267-169-96 | Indole glucosinolates | HMDB0301786 | ^9^ | Raw seeds, Unprimed mustard sprouts, MT-primed mustard sprouts |
| 16 | 3.2 | Glucobrassicin | 447  M-H | [C_16_H_20_N_2_O_9_S_2_](https://pubchem.ncbi.nlm.nih.gov/#query=C16H20N2O9S2) | 267-285-97 | Indole glucosinolates | HMDB0030243 | ^9^ | Unprimed mustard sprouts, MT-primed mustard sprouts |
| 17 | 3.4 | Hydroxyquinoline | 144.3  M-H | C_9_H_7_NO | 116-115-85 | Hydroquinolones | HMDB0246466 | ^12^ | Raw seeds, Unprimed mustard sprouts, MT-primed mustard sprouts |
| 18 | 3.6 | Acetyl tyrosine | 224.3  M+H | C_11_H_13_NO_4_ | 182-136-119 | Amino acids | HMDB0000866 | ^4^ | Raw seeds, Unprimed mustard sprouts, MT-primed mustard sprouts |
| 19 | 3.9 | Salicylic acid-O-hexoside | 299.2  M-H | C_13_H_16_O_8_ | 137-93 | Phenolic acids | HMDB0041271 | ^13^ | Unprimed mustard sprouts, MT-primed mustard sprouts |
| 20 | 4.2 | Hydroxybenzoic acid | 137.2  M-H | C_7_H_6_O_3_ | 93 | Phenolic acids | HMDB0000500 | ^1^ | Unprimed mustard sprouts, MT-primed mustard sprouts |
| 21 | 4.5 | Syringic acid | 197.01  M-H | C_9_H_10_O_5_ | 182-167-153-138 | Phenolic acids | HMDB0002085 | ^14^ | Unprimed mustard sprouts, MT-primed mustard sprouts |
| 22 | 4.8 | Sinapic acid | 225.4  M+H | C_11_H_12_O_5_ | 179 | Phenolic acids | HMDB0032616 | ^14^ | Unprimed mustard sprouts, MT-primed mustard sprouts |
| 23 | 5.4 | Vanillic acid | 167.3  M-H | C_8_H_8_O_4_ | 123-109 | Phenolic acids | HMDB0000484 | ^13^ | Raw seeds, MT-primed mustard sprouts |
| 24 | 5.8 | Coumaric acid | 163.2  M-H | C_9_H_8_O_3_ | 135 | Phenolic acids | HMDB0001713 | ^13^ | Raw seeds, Unprimed mustard sprouts, MT-primed mustard sprouts |
| 25 | 6.1 | Valyl-Phenylalanine | 265.2  M+H | C_14_H_20_N_2_O_3_ | 166-121-118 | Dipeptides | HMDB0029134 | ^15^ | Raw seeds, Unprimed mustard sprouts, MT-primed mustard sprouts |
| 26 | 6.2 | Sinapine | 310.3  M+H | [C_16_H_24_NO_5_^+^](https://pubchem.ncbi.nlm.nih.gov/#query=C16H24NO5+) | 251-207-175-147 | Phenolic acid derivatives | HMDB0029379 | ^16^ | Unprimed mustard sprouts, MT-primed mustard sprouts |
| 27 | 6.3 | Coumaroyl tartaric acid | 295.3  M-H | [C_13_H_12_O_8_](https://pubchem.ncbi.nlm.nih.gov/#query=C13H12O8) | 161-135 | Phenolic acids | HMDB0029783 | ^16^ | Raw seeds, Unprimed mustard sprouts, MT-primed mustard sprouts |
| 28 | 6.4 | Ferulic acid* | 193.2  M-H | [C_10_H_10_O_4_](https://pubchem.ncbi.nlm.nih.gov/#query=C10H10O4) | 179-135 | Phenolic acids | HMDB0029200 | ….. | Unprimed mustard sprouts, MT-primed mustard sprouts |
| 29 | 6.6 | Dihydrosinapic acid | 227.2  M+H | [C_11_H_14_O_5_](https://pubchem.ncbi.nlm.nih.gov/#query=C11H14O5) | 179-135-161 | Phenolic acids | HMDB0041727 | ^17^ | Raw seeds, Unprimed mustard sprouts, MT-primed mustard sprouts |
| 30 | 7.6 | Chlorogenic acid (3-O-Caffeoylquinic acid) | 353.2  M-H | C_16_H_18_O_9_ | 179-191-135 | Phenolic acids | HMDB0003164 | ^18^ | Unprimed mustard sprouts, MT-primed mustard sprouts |
| 31 | 7.2 | Tryptamine | 161.2  M+H | C_10_H_12_N_2_ | 145-117 | Amino acid derivatives | HMDB0000303 | ^19^ | Unprimed mustard sprouts, MT-primed mustard sprouts |
| 32 | 7.3 | Isorhapontigenin | 259.3  M+H | C₁₅H₁₄O₄ | 241-185-157 | Stilbenoids | HMDB0303171 | ^20^ | Unprimed mustard sprouts, MT-primed mustard sprouts |
| 33 | 7.4 | Ellagic acid | 301.2  M-H | C_14_H_6_O_8_ | 257-229-185 | Phenolic acids | HMDB0002899 | ^21^ | Raw seeds, Unprimed mustard sprouts, MT-primed mustard sprouts |
| 34 | 7.8 | [Astringin](https://pubchem.ncbi.nlm.nih.gov/compound/astringin) | 407.4  M+H | [C_20_H_22_O_9_](https://pubchem.ncbi.nlm.nih.gov/#query=C20H22O9) | 245-134 | Stilbenoids | HMDB0002434 | ^22^ | Unprimed mustard sprouts, MT-primed mustard sprouts |
| 35 | 8.2 | Catechin | 289.3  M-H | C_15_H_14_O_6_ | 245-205-139 | Flavonoids | HMDB0001871 | ^23^ | Raw seeds, Unprimed mustard sprouts, MT-primed mustard sprouts |
| 36 | 8.3 | Pterostilbene | 257.3  M+H | C_16_H_16_O_3_ | 242-227-197-137 | Stilbenoids | HMDB0303379 | ^24^ | Unprimed mustard sprouts, MT-primed mustard sprouts |
| 37 | 8.5 | Syringetin-3-O-glucoside | 507.3  M-H | [C_23_H_24_O_13_](https://pubchem.ncbi.nlm.nih.gov/#query=C23H24O13) | 346-329-314-261-165-149 | Flavonoids | HMDB0304565 | ^25^ | Unprimed mustard sprouts, MT-primed mustard sprouts |
| 38 | 8.6 | Peonidin-3-O-glucoside | 464.3  M+H | C_22_H_23_O_11_ | 301-286-258-213-201 | Flavonoids | HMDB0013689 | ^26^ | MT-primed mustard sprouts |
| 39 | 8.8 | Methyl Coumarin | 161.2  M+H | C_10_H_8_O_2_ | 147-118 | Coumarins | HMDB0032394 | ^27^ | Unprimed mustard sprouts, MT-primed mustard sprouts |
| 40 | 9.2 | Luteolin-O-glucoside | 449.3  M+H | C_21_H_20_O_11_ | 285-219-147-73 | Flavonoids | HMDB0038468 | ^28^ | Unprimed mustard sprouts, MT-primed mustard sprouts |
| 41 | 9.5 | Sinalbin A | 279.2  M-H | C_14_H_19_NO_9_S_2_ | 133-124-108 | Alkylindoles | HMDB0036650 | ^29^ | Raw seeds, Unprimed mustard sprouts |
| 42 | 9.6 | Quercetin* | 301.2  M-H | C_15_H_10_O_7_ | 255-179-151 | Flavonoids | HMDB0005794 | …….. | Raw seeds, Unprimed mustard sprouts, MT-primed mustard sprouts |
| 43 | 9.9 | Kaempferol | 285.3  M-H | C_15_H_10_O_6_ | 257-217-151 | Flavonoids | HMDB0005801 | ^30^ | Unprimed mustard sprouts, MT-primed mustard sprouts |
| 44 | 10.3 | Daidzein | 253.3  M-H | C_15_H_10_O_4_ | 237-151-135 | Flavonoids | HMDB0003312 | ^31^ | Unprimed mustard sprouts, MT-primed mustard sprouts |
| 45 | 10.6 | Isorhamnetin | 315.3  M-H | C_16_H_12_O_7_ | 300-285-163 | Flavonoids | HMDB0002655 | ^14^ | Raw seeds |
| 46 | 11.1 | Feruloyl tryptamine | 337.3  M+H | C_20_H_20_N_2_O_3_ | 195-181-161-137 | Phenolic acid derivatives | HMDB0041519 | ^32^ | Unprimed mustard sprouts, MT-primed mustard sprouts |
| 47 | 11.4 | Nonanedioic acid (Azelaic acid) | 187.3  M-H | C_9_H_16_O_4_ | 143 | Fatty acids | HMDB0000784 | ^1^ | Raw seeds, Unprimed mustard sprouts, MT-primed mustard sprouts |
| 48 | 11.7 | Hydroxy lauric acid | 217.2  M+H | C_12_H_24_O_3_ | 201-157 | Fatty acid “oxylipins” | HMDB0010728 | ^33^ | Raw seeds, Unprimed mustard sprouts, MT-primed mustard sprouts |
| 49 | 12.2 | Dodecanoic acid (Lauric acid) | 201.2  M+H | C_12_H_24_O_2_ | 157 | Fatty acids | HMDB0000638 | ^15^ | Raw seeds, Unprimed mustard sprouts, MT-primed mustard sprouts |
| 50 | 13.4 | Carnosic acid | 333.3  M+H | C_20_H_28_O_4_ | 279-246 | Terpenoids | HMDB0002358 | ^34^ | Raw seeds |
| 51 | 13.7 | Myristic acid | 229.2  M+H | C_14_H_28_O_2_ | 185-131-75 | Fatty acids | HMDB0000806 | ^35^ | Raw seeds, Unprimed mustard sprouts, MT-primed mustard sprouts |
| 52 | 15.3 | 1-palmitoyl-sulfoquinovopyranosylglycerol SQMG (16:0) | 555.6  M-H | [C_25_H_48_O_11_S](https://pubchem.ncbi.nlm.nih.gov/#query=C25H48O11S) | 300-255-81 | Sulfoglycolipids | HMDB0007853 | ^36^ | Raw seeds, Unprimed mustard sprouts, MT-primed mustard sprouts |
| 53 | 14.8 | PE (12:0/16:0) | 636.2  M+H | [C_33_H_66_NO_8_P](https://pubchem.ncbi.nlm.nih.gov/#query=C33H66NO8P) | 454-436-399-381-257-213 | Phospholipids | HMDB0001852 | ^37^ | Raw seeds, Unprimed mustard sprouts, MT-primed mustard sprouts |
| 54 | 15.8 | PG (14:0/14:0) | 665.8  M-H | [C_34_H_67_O_10_P](https://pubchem.ncbi.nlm.nih.gov/#query=C34H67O10P) | 648-438-420-227-167 | Phosphoglycerols | HMDB0007866 | ^12^ | Raw seeds, Unprimed mustard sprouts, MT-primed mustard sprouts |
| 55 | 16.7 | LysoPC (16:0) | 496.4  M+H | C_24_H_50_NO_7_P | 478-259-257-241-213 | Phospholipids  “Phosphocholines” | HMDB0010382 | ^12^ | Raw seeds |
| 56 | 17.6 | Hydroxy palmitic acid | 271.3  M-H | C_16_H_32_O_3_ | 255-211 | Fatty acids”oxylipins” | HMDB0031057 | ^38^ | Raw seeds |
| 57 | 18.2 | Stearidionoyl-Glycerol | 351.2  M+H | C_21_H_34_O_4_ | 277-233-162 | Fatty acid glycerides | HMDB0244009 | ^39^ | Raw seeds, Unprimed mustard sprouts, MT-primed mustard sprouts |
| 58 | 18.8 | Stearidionic acid | 277.3  M+H | C_18_H_28_O_2_ | 255- 233-162 | Fatty acids | HMDB0006547 | ^38^ | Unprimed mustard sprouts, MT-primed mustard sprouts |
| 59 | 19.3 | Hydroxy linolenic Acid | 293.2  M-H | C_18_H_30_O_3_ | 277-233-59 | Fatty acid “oxylipins” | HMDB0031103 | ^40^ | Raw seeds, Unprimed mustard sprouts, MT-primed mustard sprouts |
| 60 | 19.5 | LysoPC 18:3 | 518.2  M+H | C_26_H_48_NO_7_P | 503-277-257-235-239-59 | Phospholipids  “Phosphocholines” | HMDB0010388 | ^40^ | Unprimed mustard sprouts, MT-primed mustard sprouts |
| 61 | 19.8 | Linolenoyl-glycerol | 353.4  M+H | C_21_H_36_O_4_ | 279-235-59 | Lipids (fatty acid glycerides) | HMDB0011559 | ^18^ | Raw seeds, Unprimed mustard sprouts, MT-primed mustard sprouts |
| 62 | 20.4 | Beta-tocopherol | 415.2  M-H | C_28_H_48_O_2_ | 394-151 | Diterpenoids “tocopherols” | HMDB0006335 | ^41^ | Raw seeds, Unprimed mustard sprouts, MT-primed mustard sprouts |
| 63 | 20.9 | Linolenic acid | 277.2  M-H | C_18_H_30_O_2_ | 235-59 | Lipids (fatty acids) | HMDB0003073 | ^12^ | Unprimed mustard sprouts, MT-primed mustard sprouts |
| 64 | 21.3 | Linoleoyl-glycerol | 355.2  M+H | C_21_H_38_O_4_ | 281-241-59 | Lipids (fatty acid glycerides) | [HMDB0245187](https://hmdb.ca/metabolites/HMDB0245187) | ^18^ | Unprimed mustard sprouts, MT-primed mustard sprouts |
| 65 | 22.3 | Eicosapentaenoic acid | 303.2  M+H | C_20_H_30_O_2_ | 259-110 | fatty acids | HMDB0001999 | ^42^ | Raw seeds, Unprimed mustard sprouts, MT-primed mustard sprouts |
| 66 | 23.5 | Ricinoleic acid | 299.3  M+H | C_18_H_34_O_3_ | 283-239-126-59 | fatty acids | HMDB0034297 | ^43^ | Raw seeds, Unprimed mustard sprouts, MT-primed mustard sprouts |
| 67 | 24.6 | Hydroxy arachidonic acid | 319.2  M-H | C_20_H_32_O_3_ | 303-287-259 | Fatty acid “oxylipins” | HMDB0060101 | ^44^ | Unprimed mustard sprouts, MT-primed mustard sprouts |
| 68 | 25.2 | Stearic acid | 283.4  M-H | C_18_H_36_O_2_ |  | Lipids (fatty acid glycerides) | HMDB0000827 | ^6^ | Raw seeds, Unprimed mustard sprouts, MT-primed mustard sprouts |
| 69 | 26.3 | Stigmasterol* | 413.2  M+H | C_29_H_48_O | 399-277-165 | Sterols | HMDB0000937 | …… | Raw seeds, Unprimed mustard sprouts, MT-primed mustard sprouts |
| 70 | 27.2 | Eicosenoic acid | 309.2  M-H | C_20_H_38_O_2_ | 291-265-141-71 | Fatty acids | HMDB0002231 | ^45^ | Raw seeds, Unprimed mustard sprouts, MT-primed mustard sprouts |
| 71 | 28.5 | Erucic acid | 339.2  M+H | C_22_H_42_O_2_ | 321-295-181 | Fatty acids | HMDB0002068 | ^46^ | Raw seeds, Unprimed mustard sprouts, MT-primed mustard sprouts |

*** Identified by comparison with external standards**

**Table S2: The relative abundances of the detected metabolites across the treatment groups (unprimed and MT-primed germinated seeds)**

| **Compound Name** | **Germinated Seeds MS (G)** | **MT-Primed Seeds MS (MT)** | **log2(FC)** | **Linear FC** |
| --- | --- | --- | --- | --- |
| Hydroxybenzoic acid | (3.25 ± 0.42) × 10^5 | (5.49 ± 0.74) × 10^7 | 7.4 | 168.68 |
| Serine | (8.75 ± 1.14) × 10^6 | (2.10 ± 0.28) × 10^5 | -5.38 | 0.02 |
| Dihydrosinapic acid | (5.33 ± 0.69) × 10^5 | (3.79 ± 0.51) × 10^7 | 6.15 | 71.16 |
| Isorhamnetin | (3.01 ± 0.39) × 10^7 | (8.71 ± 1.17) × 10^8 | 4.86 | 28.95 |
| Salicylic acid-O-hexoside | (1.05 ± 0.14) × 10^6 | (3.62 ± 0.49) × 10^7 | 5.11 | 34.49 |
| LysoPC (16:0) | (2.60 ± 0.34) × 10^6 | (8.69 ± 1.16) × 10^7 | 5.06 | 33.42 |
| Leucine | (8.05 ± 1.05) × 10^8 | (4.97 ± 0.67) × 10^7 | -4.02 | 0.06 |
| Erucic acid | (2.37 ± 0.31) × 10^6 | (3.76 ± 0.50) × 10^7 | 3.99 | 15.91 |
| Syringetin-3-O-glucoside | (1.85 ± 0.24) × 10^6 | (2.67 ± 0.36) × 10^7 | 3.85 | 14.46 |
| Eicosenoic acid | (1.19 ± 0.16) × 10^6 | (1.68 ± 0.22) × 10^7 | 3.81 | 14.05 |
| Gluconapin | (8.43 ± 1.09) × 10^6 | (9.11 ± 1.22) × 10^7 | 3.53 | 11.58 |
| Coumaric acid | (5.50 ± 0.72) × 10^7 | (4.63 ± 0.62) × 10^8 | 3.07 | 8.42 |
| Hydroxy palmitic acid | (6.35 ± 0.83) × 10^7 | (4.90 ± 0.66) × 10^8 | 2.95 | 7.73 |
| Isorhapontigenin | 0.00 ± 0.00 | (1.10 ± 0.15) × 10^7 | 2.59 | 6.01 |
| Glucotropaeolin | 0.00 ± 0.00 | (1.10 ± 0.15) × 10^7 | 2.89 | 7.41 |
| Acetyl tyrosine | 0.00 ± 0.00 | (1.56 ± 0.21) × 10^7 | 2.59 | 6.02 |
| Linoleoyl glycerol | (5.50 ± 0.72) × 10^7 | (2.38 ± 0.32) × 10^8 | 2.12 | 4.34 |
| Ferulic acid | (2.08 ± 0.27) × 10^7 | (8.94 ± 1.20) × 10^7 | 3.1 | 8.59 |
| Eicosapentaenoic acid | (1.34 ± 0.17) × 10^8 | (5.53 ± 0.74) × 10^8 | 2.35 | 5.1 |
| Syringic acid | (4.27 ± 0.56) × 10^7 | (1.64 ± 0.22) × 10^8 | 3.83 | 14.21 |
| Hydroxy glucobrassicin | (1.31 ± 0.17) × 10^7 | (4.95 ± 0.66) × 10^7 | 2.08 | 4.22 |
| Linolenoyl glycerol | (1.44 ± 0.19) × 10^8 | (5.44 ± 0.73) × 10^8 | 3.77 | 13.61 |
| LysoPC 18:3 | (1.23 ± 0.16) × 10^7 | (4.47 ± 0.60) × 10^7 | 3.65 | 12.56 |
| Ellagic acid | (1.72 ± 0.22) × 10^7 | (5.72 ± 0.77) × 10^7 | 2.52 | 5.73 |
| PG (12:0/16:0) | (4.67 ± 0.61) × 10^7 | (1.54 ± 0.21) × 10^8 | 2.29 | 4.9 |
| Quercetin | (2.29 ± 0.30) × 10^7 | (7.50 ± 1.01) × 10^7 | 3.28 | 9.68 |
| Methyl Coumarin | (4.21 ± 0.55) × 10^7 | (1.35 ± 0.18) × 10^8 | 3.2 | 9.22 |
| Feruloyl tryptamine | (3.77 ± 0.49) × 10^7 | (1.19 ± 0.16) × 10^8 | 3.15 | 8.9 |
| Sinapic acid | (6.85 ± 0.89) × 10^7 | (1.95 ± 0.26) × 10^8 | 2.84 | 7.17 |
| Hydroxy arachidonic acid | (1.94 ± 0.25) × 10^8 | (5.50 ± 0.74) × 10^8 | 2.8 | 6.96 |
| Sinigrin | (5.89 ± 0.77) × 10^7 | (1.64 ± 0.22) × 10^8 | 2.6 | 6.06 |
| Pterostilbene | (7.69 ± 1.00) × 10^7 | (1.99 ± 0.27) × 10^8 | 2.84 | 7.17 |
| Chlorogenic acid | (1.56 ± 0.20) × 10^7 | (3.75 ± 0.50) × 10^7 | 2.5 | 5.66 |
| Phenylalanine | (3.89 ± 0.51) × 10^8 | (9.31 ± 1.25) × 10^8 | 2.3 | 4.92 |
| Kaempferol | (2.25 ± 0.29) × 10^7 | (4.67 ± 0.63) × 10^7 | 2.07 | 4.2 |
| Hydroxy linolenic acid | (3.40 ± 0.44) × 10^8 | (6.95 ± 0.93) × 10^8 | 2.03 | 4.08 |

***** **Values are expressed as mean peak intensity** $\boldsymbol{\pm}$ **standard deviation (**$\boldsymbol{n=5}$**). Fold change (FC) is calculated as** $\text{Mean}_{\text{MT-Primed}}\boldsymbol{/}\text{Mean}_{\text{Germinated}}$**. On a** $\log_{2} \left( \text{FC} \right)$**scale, a fold change greater than 2 equals an actual fold change greater than 4, and less than 0.5 corresponds to a** $\log_{2} \left( \text{FC} \right)$**value below -1 (2^-1^ = 0.5). Criteria requiring a** $\log_{2} \left( \text{FC} \right)$**fold change greater than 2 or less than -1 are very strict, filtering for massive metabolic shifts**

**Figure S1: Base peak chromatograms (BPCs) for unprimed germinated (control) and MT-primed mustard samples, captured in both positive-ion and negative-ion modes.**

| ***Acetylcholine esterase (AChE)*** | ***Monoamine oxidase (MAO-B)*** |
| --- | --- |
| **MS (MT)**   | **MS (MT)**   |
| **MS (G)**   | **MS (G)**   |
| **MS (raw)**   | **MS (raw)**   |
| **Physostigmine**   | **Pargyline**   |
|  |  |

References

1.	Ghallab, D. S., Mohyeldin, M. M., Shawky, E., Metwally, A. M. & Ibrahim, R. S. Chemical profiling of Egyptian propolis and determination of its xanthine oxidase inhibitory properties using UPLC–MS/MS and chemometrics. *Lwt* **136**, 110298 (2021).

2.	Song, L., Morrison, J. J., Botting, N. P. & Thornalley, P. J. Analysis of glucosinolates, isothiocyanates, and amine degradation products in vegetable extracts and blood plasma by LC–MS/MS. *Anal. Biochem.* **347**, 234–243 (2005).

3.	Kelley, J. M., Hughes, L. B. & Bridges, S. L. Does gamma-aminobutyric acid (GABA) influence the development of chronic inflammation in rheumatoid arthritis? *J. Neuroinflammation* **5**, 1–5 (2008).

4.	Zhang, P. *et al.* Revisiting fragmentation reactions of protonated α-amino acids by high-resolution electrospray ionization tandem mass spectrometry with collision-induced dissociation. *Sci. Rep.* **9**, 1–10 (2019).

5.	Ghallab, D. S., Ibrahim, R. S., Ghareeb, D. A. & El Newehy, N. M. Multi-omics for unveiling potential antidiabetic markers from red, green and black mung beans using NIR-UPLC-MS/MS multiplex approach. *Sci. Rep.* **15**, 1–18 (2025).

6.	Ghallab, D. S., Shawky, E., Ibrahim, R. S. & Mohyeldin, M. M. Comprehensive metabolomics unveil the discriminatory metabolites of some Mediterranean Sea marine algae in relation to their cytotoxic activities. *Sci. Rep.* **12**, 8094 (2022).

7.	Mohn, T., Cutting, B., Ernst, B. & Hamburger, M. Extraction and analysis of intact glucosinolates—A validated pressurized liquid extraction/liquid chromatography–mass spectrometry protocol for Isatis tinctoria, and qualitative analysis of other cruciferous plants. *J. Chromatogr. A* **1166**, 142–151 (2007).

8.	Kim, H. W. *et al.* Identification and quantification of glucosinolates in Korean leaf mustard germplasm (Brassica juncea var. integrifolia) by liquid chromatography–electrospray ionization/tandem mass spectrometry. *Eur. Food Res. Technol.* **242**, 1479–1484 (2016).

9.	Hwang, I. M., Park, B., Dang, Y. M., Kim, S.-Y. & Seo, H. Y. Simultaneous direct determination of 15 glucosinolates in eight Brassica species by UHPLC-Q-Orbitrap-MS. *Food Chem.* **282**, 127–133 (2019).

10.	Dezena, R. M. B., Mauri, R., Barros, F. A. P. & Rosa, P. C. P. Characterization of indole-3-acetic acid in cambial zone tissue by ultra-performance liquid chromatography-tandem mass spectrometry. *J. Appl. Biol. Biotechnol. Vol* **7**, 64–70 (2019).

11.	Li, Z. *et al.* Characterization of glucosinolates in 80 broccoli genotypes and different organs using UHPLC-Triple-TOF-MS method. *Food Chem.* **334**, 127519 (2021).

12.	Ghallab, D. S., Nasr, S. A., Ghareeb, D. A. & Abd El Hafez, M. S. M. Cooking and salting impacts on sea cucumber (Holothuria atra) metabolome and their entanglements on anticoagulant potential as revealed by UPLC-QqQ-MS/MS analysis and chemometrics. *J. Food Compos. Anal.* 106911 (2024).

13.	Mena, P. *et al.* Phytochemical profiling of flavonoids, phenolic acids, terpenoids, and volatile fraction of a rosemary (Rosmarinus officinalis L.) extract. *Molecules* **21**, 1576 (2016).

14.	Alberti, Á. LC-ESI-MS/MS methods in profiling of flavonoid glycosides and phenolic acids in traditional medicinal plants: Sempervivum tectorum L. and Corylus avellana L. at (2014).

15.	Dawczynski, C., Schubert, R. & Jahreis, G. Amino acids, fatty acids, and dietary fibre in edible seaweed products. *Food Chem.* **103**, 891–899 (2007).

16.	Guan, H., Lin, Q., Ma, C., Ju, Z. & Wang, C. Metabolic profiling and pharmacokinetic studies of sinapine thiocyanate by UHPLC-Q/TOF-MS and UHPLC-MS/MS. *J. Pharm. Biomed. Anal.* **207**, 114431 (2022).

17.	Buiarelli, F., Coccioli, F., Merolle, M., Jasionowska, R. & Terracciano, A. Identification of hydroxycinnamic acid–tartaric acid esters in wine by HPLC–tandem mass spectrometry. *Food Chem.* **123**, 827–833 (2010).

18.	Ghallab, D. S. & Ghareeb, D. A. UPLC-MS/MS-based metabolomics and chemometrics unveil metabolic patterns and their regulatory pathways over the course of pumpkin seeds (Cucurbita maxima) germination. *Sci. Hortic. (Amsterdam).* **337**, 113563 (2024).

19.	Schüller, M., McQuade, T. A.-P., Bergh, M. S.-S., Pedersen-Bjergaard, S. & Øiestad, E. L. Determination of tryptamine analogs in whole blood by 96-well electromembrane extraction and UHPLC-MS/MS. *Talanta Open* **7**, 100171 (2023).

20.	Dai, Y. *et al.* Pre-clinical pharmacokinetic and metabolomic analyses of isorhapontigenin, a dietary resveratrol derivative. *Front. Pharmacol.* **9**, 753 (2018).

21.	Ghallab, D. S. *et al.* Exploring the anti-inflammatory bioactive metabolites of some marine algae through integration of metabolomics, network pharmacology and molecular docking analyses. *Food Biosci.* **50**, 102091 (2022).

22.	Zhuang, J. *et al.* Evaluation of astringent taste of green tea through mass spectrometry-based targeted metabolic profiling of polyphenols. *Food Chem.* **305**, 125507 (2020).

23.	Dutschke, J., Suchowski, M. & Pietsch, J. Simultaneous determination of selected catechins and pyrogallol in deer intoxications by HPLC-MS/MS. *J. Chromatogr. B* **1180**, 122886 (2021).

24.	Wang, H. *et al.* Comprehensive Analysis of Pterostilbene Metabolites in Vivo and in Vitro Using a UHPLC-Q-Exactive Plus Mass Spectrometer with Multiple Data-Mining Methods. *ACS Omega* **7**, 38561–38575 (2022).

25.	Flamini, R., De Rosso, M. & Bavaresco, L. Study of grape polyphenols by liquid chromatography‐high‐resolution mass spectrometry (UHPLC/QTOF) and suspect screening analysis. *J. Anal. Methods Chem.* **2015**, 350259 (2015).

26.	Vega, E. N., Fernández-Ruiz, V., Sánchez-Mata, M. C., Cámara, M. & Morales, P. A Rapid and Simple UHPLC-DAD Method for Individual Anthocyanin Analysis: Optimization and Validation for Wild Mediterranean Berries and Fruit Samples. *Food Anal. Methods* **18**, 471–486 (2025).

27.	Dugrand, A. *et al.* Coumarin and furanocoumarin quantitation in citrus peel via ultraperformance liquid chromatography coupled with mass spectrometry (UPLC-MS). *J. Agric. Food Chem.* **61**, 10677–10684 (2013).

28.	Hussain, A. *et al.* Determination of total phenolic, flavonoid, carotenoid, and mineral contents in peel, flesh, and seeds of pumpkin (Cucurbita maxima). *J. Food Process. Preserv.* **45**, e15542 (2021).

29.	Popova, I. E. & Morra, M. J. Sinigrin and sinalbin quantification in mustard seed using high performance liquid chromatography–time-of-flight mass spectrometry. *J. Food Compos. Anal.* **35**, 120–126 (2014).

30.	Nada, A. A. *et al.* An integrated strategy for chemical, biological and palynological standardization of bee propolis. *Microchem. J.* **182**, 107923 (2022).

31.	Zhao, W. *et al.* Rapid screening and identification of daidzein metabolites in rats based on UHPLC-LTQ-orbitrap mass spectrometry coupled with data-mining technologies. *Molecules* **23**, 151 (2018).

32.	Li, K. & Wang, W. An Optimized and Sensitive UHPLC‐ESI‐Q‐TOF/HRMS Demonstrated the Dynamic Profiling of Allantoin, N‐trans‐Feruloyl‐3‐Methoxytyramine, and N‐p‐Coumaroyltyramine from Portulaca oleracea L. In Vivo in Rats. *J. Sep. Sci.* **48**, e70055 (2025).

33.	Griffiths, W. J. Tandem mass spectrometry in the study of fatty acids, bile acids, and steroids. *Mass Spectrom. Rev.* **22**, 81–152 (2003).

34.	Farhadi, F., Baradaran Rahimi, V., Mohamadi, N. & Askari, V. R. Effects of rosmarinic acid, carnosic acid, rosmanol, carnosol, and ursolic acid on the pathogenesis of respiratory diseases. *BioFactors* **49**, 478–501 (2023).

35.	Divito, E. B., Davic, A. P., Johnson, M. E. & Cascio, M. Electrospray ionization and collision induced dissociation mass spectrometry of primary fatty acid amides. *Anal. Chem.* **84**, 2388–2394 (2012).

36.	Ibrahim, R. M. *et al.* Unveiling the functional components and antivirulence activity of mustard leaves using an LC-MS/MS, molecular networking, and multivariate data analysis integrated approach. *Food Res. Int.* **168**, 112742 (2023).

37.	Zhang, D., Duan, X. & Sun, H. Phospholipidomics and quantum chemistry calculation unravel the changes in phospholipid molecules of flaxseed oil during roasting. *Food Chem.* **404**, 134579 (2023).

38.	Gowda, S. G. B. *et al.* Identification of short‐chain fatty acid esters of hydroxy fatty acids (SFAHFAs) in a murine model by nontargeted analysis using ultra‐high‐performance liquid chromatography/linear ion trap quadrupole‐Orbitrap mass spectrometry. *Rapid Commun. Mass Spectrom.* **34**, e8831 (2020).

39.	Ray, J., Smith, K. W., Bhaggan, K., Nagy, Z. K. & Stapley, A. G. F. Characterisation of high 1, 3‐distearoyl‐2‐oleoyl‐sn‐glycerol content stearins produced by acidolysis of high oleic sunflower oil with stearic and palmitic acids. *Eur. J. Lipid Sci. Technol.* **116**, 532–547 (2014).

40.	Ghallab, D. S., Ghareeb, D. A. & Goda, D. A. Integrative metabolomics and chemometrics depict the metabolic alterations of differently processed red kidney beans (Phaseolus vulgaris L.) and in relation to in-vitro anti-diabetic efficacy. *Food Res. Int.* 114786 (2024).

41.	Ansolin, M., de Souza, P. T., de Almeida Meirelles, A. J. & Batista, E. A. C. Tocopherols and tocotrienols: An adapted methodology by UHPLC/MS without sample pretreatment steps. *Food Anal. Methods* **10**, 2165–2174 (2017).

42.	Li, X. *et al.* Extraction and purification of eicosapentaenoic acid and docosahexaenoic acid from microalgae: A critical review. *Algal Research* vol. 43 at https://doi.org/10.1016/j.algal.2019.101619 (2019).

43.	Choi, G. H. *et al.* Quantitative analyses of ricinoleic acid and ricinine in Ricinus communis extracts and its biopesticides. *J. Appl. Biol. Chem.* **59**, 165–169 (2016).

44.	Edpuganti, V. & Mehvar, R. UHPLC–MS/MS analysis of arachidonic acid and 10 of its major cytochrome P450 metabolites as free acids in rat livers: Effects of hepatic ischemia. *J. Chromatogr. B* **964**, 153–163 (2014).

45.	Yang, B., Yang, H., Chen, F., Hua, Y. & Jiang, Y. Phytochemical analyses of Ziziphus jujuba Mill. var. spinosa seed by ultrahigh performance liquid chromatography-tandem mass spectrometry and gas chromatography-mass spectrometry. *Analyst* **138**, 6881–6888 (2013).

46.	Crescenzi, M. A., Cerulli, A., Montoro, P. & Piacente, S. Metabolite profiling for typization of “rucola della piana del sele”(PGI), Eruca sativa, through UHPLC-Q-Exactive-Orbitrap-MS/MS analysis. *Foods* **12**, 3384 (2023).
